# NESSTI: Norms for Environmental Sound Stimuli

**DOI:** 10.1371/journal.pone.0073382

**Published:** 2013-09-04

**Authors:** Julia Hocking, Ilvana Dzafic, Maria Kazovsky, David A. Copland

**Affiliations:** 1 Centre for Advanced Imaging, University of Queensland, St Lucia, Australia; 2 Centre for Clinical Research, University of Queensland, Herston, Australia; 3 School of Health & Rehabilitation Sciences, University of Queensland, St Lucia, Australia; Charité University Medicine Berlin, Germany

## Abstract

In this paper we provide normative data along multiple cognitive and affective variable dimensions for a set of 110 sounds, including living and manmade stimuli. Environmental sounds are being increasingly utilized as stimuli in the cognitive, neuropsychological and neuroimaging fields, yet there is no comprehensive set of normative information for these type of stimuli available for use across these experimental domains. Experiment 1 collected data from 162 participants in an on-line questionnaire, which included measures of identification and categorization as well as cognitive and affective variables. A subsequent experiment collected response times to these sounds. Sounds were normalized to the same length (1 second) in order to maximize usage across multiple paradigms and experimental fields. These sounds can be freely downloaded for use, and all response data have also been made available in order that researchers can choose one or many of the cognitive and affective dimensions along which they would like to control their stimuli. Our hope is that the availability of such information will assist researchers in the fields of cognitive and clinical psychology and the neuroimaging community in choosing well-controlled environmental sound stimuli, and allow comparison across multiple studies.

## Introduction

The critical importance of utilizing stimuli that are controlled along multiple dimensions when carrying out cognitive testing of object-related processing is well established. A large number of studies in both behavioral and neuroimaging experimental domains have been dedicated to the identification of variables that can influence the naming and recognition of visual objects and words, and these variables have been identified across multiple languages [1], [2], [3]. In contrast, far less is known about the variables influencing the recognition and identification of non-linguistic auditory inputs, and normative studies to date have tended to focus on either small numbers of sound stimuli, stimuli with long and/or varying lengths, or synthetic sounds. Norms appropriate to a particular paradigm are essential because they provide key data on the extraneous variables that might confound an empirical outcome. Only by understanding and controlling these variables are we able to measure the process of interest. In this paper we describe a large set of natural environmental sounds designed for use in both the behavioral and neuroimaging experimental domains, and provide a set of measurements along multiple dimensions that may influence and assist in the selection of appropriate environmental sound stimuli. Understanding the variables that influence recognition and identification may also help in the interpretation of stimulus-specific differences that could be evoked during processing of environmental sounds.

Since the seminal work of Snodgrass and Vanderwart [4], multiple visual object norming studies have been published (see [5] for a review). These studies have highlighted the range of cognitive, neuroanatomical and physiological variables that influence the perception, recognition or naming of visual concepts, and have enabled researchers to not only control for these potential confounding factors, but also investigate these factors in their own right. For example, the effect of color [6], age of acquisition [7], visual structural similarity [8], linguistic frequency [9] and so forth have been examined and have been demonstrated to have strong effects on both behavioral performance and imaging data. Systematic control over these variables is thus considered a prerequisite for well-controlled experiments using pictorial and linguistic stimuli in both the behavioral and neuroimaging domains.

From the perspective of neuropsychology, the importance of controlling concept-related variables has also been highlighted. Reports of category-specific semantic impairments that disappeared when living and nonliving categories were matched along the dimensions of familiarity, frequency and visual complexity clearly illustrate the relevance of controlling these variables [10], [11], [12]. Independent of the processing level at which this difference between impaired and intact performance may have occurred, e.g., perceptual, semantic [13], these findings demonstrate the critical impact that a change in object-related variables can have on understanding visual object processing and functional neuroanatomy in the patient population.

### Environmental Sound Norming Studies

Object knowledge is derived from the interaction of multiple senses, yet our understanding about the variables that influence human object processing stems predominantly from studies of visual words and pictures. Far less is known about the variables that influence the processing of environmental sounds, and to date there have been only a small number of studies investigating the variables that may influence the successful recognition and identification of environmental sounds that can be used as normative data. In Table 1 we have provided a summary of these studies and the variables that were measured. Although multiple different variables have been considered, only the study by Marcell, Borella, Greene, Kerr, and Rogers [14] provided a comprehensive set of measurements of naturally occurring sounds across a range of conceptual categories. However, because these sounds were of variable temporal duration, they are less suitable for use in well-controlled paradigms in the cognitive or neuroimaging domains.

**Table 1:** Review of 13 published data sets reporting environmental sound norms.

Publication	PTCP	Sounds	Access	Categories	Duration	FID	CID	RT	CT	FM	RP	CF	AF	CP	AC
**Ballas ** [**25**]	30 (Ex1)	41	N	H M MI N	0.625s	✓	–	✓	–	✓	✓	–	✓	–	✓
**Bradley, Lang ** [**37**]	100	111	Y	H A M MI N	6.0s	–	–	–	–	–	–	–	✓	–	–
**Bradley, Lang ** [**32**]	116 (Ex1)	60	Y	NR	6.0s	–	–	–	–	–	–	–	✓	–	–
	67 (Ex2)					–	–	–	–	–	–	–	✓	–	–
**Fabiani ** [**44**]	77 (Ex1)	100	Y	A H M MI T S	Variable ≤0.4s	✓	–	–	–	–	–	–	–	–	–
	41 (Ex2)					✓	–	–	–	–	–	–	–	–	–
	17 (Ex3)					✓	–	–	–	–	–	–	–	–	–
	61 (Ex4)					✓	–	–	–	–	–	–	–	–	–
**Giordano ** [**20**]	20 (Ex1)	140	N	A H M MI N S	Median 5.23s	✓	–	✓	–	–	–	–	–	–	–
	60 (Ex2)					–	–	–	–	–	–	–	–	–	✓
**Gygi ** [**45**]	4 (Ex1)	70	N*	A H M MI N	Range 0.431–3.945s	–	✓	–	–	–	–	–	–	–	✓
	8 (Ex2)					–	✓	–	–	–	–	–	–	–	✓
	8 (Ex3a)					–	✓	–	–	–	–	–	–	–	✓
	8 (Ex3b)					–	✓		–	–	–	–	–	–	✓
	8 (Ex3c)					–	✓	–	–	–	–	–	–	–	✓
**Gygi ** [**46**]	4 (Ex1)	50	N*	A H M MI N	Range 0.579–3.945s	–	–	–	–	–	–	–	–	–	✓
	17 (Ex4)					–	–	–	✓	–	–	–	–	–	–
**Lass ** [**47**]	30	40	N		NR	✓	–	–	–	–	–	✓	–	–	–
**Marcell ** [**14**]	25 (Ex1)	120	Y	H A M MI N	Range 0.137–6.083s	✓	–	–	–	✓	–	✓	✓	✓	–
	25 (Ex2)					✓	–	✓	✓	–	–	✓	–	–	–
	38 (Ex3a)					–	–	–	✓	–	–	–	–	–	–
	49 (Ex3b)					–	–	–	✓	–	–	–	–	–	–
**Saygin ** [**41**]	31	236	Y	NR	Range 0.521–4.516s	✓	–	✓	–	–	–	–	–	–	–
**Schneider ** [**29**]	56	180	Y	S	0.4s	✓	–	–	✓	✓	–	✓	✓	–	–
**Shafiro ** [**26**]	21 (Ex1)	48	N	H A M N	Mean 2.67s	–	✓	–	–	✓	–	–	–	–	–
	7 (Ex2)	40		H A M N	Range 0.1–8.89s	–	–	–	–	–	–	–	–	–	✓
**Shafiro, Gygi ** [**48**]	65	60	Y	H A M N	Variable ≤10s	–	✓	–	–	–	–	–	–	–	✓

PTCP = Number of study participants; Access = Availability of sounds for download and/or use; FID = Free identification; CID = Closed-set identification (i.e., a list of possible sounds is provided to participants for selection); RT = Response Time; CT = Categorization; FM = Familiarity; RP = Representativeness; CF = Confidence; AF = Affect; CP = Complexity; AC = Acoustic variables; NR = Not reported. Key to sound categories: H = human generated; A = animal sounds; M = manmade noises; MI = music, musical instruments; N = naturally occurring sounds (e.g., waves breaking, wind blowing); T = pure tones; S = synthesized/artificial sounds. Acoustic variables included spectrally degraded sound in [48]; N = No; Y = YES; N* = the website supporting download of these sounds is currently unavailable. Details for Experiments 2 and 3 by Gygi et al. [46] are not included because the method involved judgment of imagined sounds and not heard sounds. Measurement of physiological responses to sounds have not been included in this table.

### Variables Measured in the Current Study

When designing this study, we considered the following variables to be of primary value across multiple domains for empirical research into object-level natural sound processing: Response latencies, identification, categorization, familiarity, confidence, representativeness, affective ratings and imageability. In order to maximize the number of respondents, we utilized both an on-line questionnaire format for qualitative responses (Study 1), and a laboratory-based study for both qualitative and response times variables (Study 2). This enabled the efficient collection of a large number of responses across multiple different variables. We therefore obtained the following data:

#### Response latencies

Response times are used to measure multiple psychological variables, particularly in object naming tasks. For example, response time differences are said to index variables such as semantic interference [15], visual (structural) similarity [16], name agreement for objects with multiple names [17], or noun/verb differences in picture naming [18].

#### Identification

Naming is perhaps the most basic and most commonly utilized task investigating the processing, organization and retrieval of object knowledge. This is exemplified by the studies listed in Table 1. Naming accuracy, name agreement, naming response times and errors during naming have all been used to inform models of the organization of conceptual processing networks at theoretical, psychological and physiological levels. Determining the most common name for an item is thus essential for maximizing control over lexical characteristics across modalities.

#### Categorization

Since the first reports of patients with naming deficits that dissociate across object domains [19], the fact that brain damage can result in impaired naming of one category of items (e.g., animals) in the context of intact naming for an alternative category (e.g., tools), has provided a rich source of information on the possible ways in which conceptual knowledge may be organized. Differences in the cognitive processing of environmental sounds along the category dimension have been explicitly investigated in the behavioral [20], [21], neuropsychological [22] and neuroimaging fields [23], [24]. These included variations between the processing of living versus manmade sounds, action-related sounds versus animal vocalizations, or the presence versus absence of human sounds in urban environments. Despite category membership of environmental sounds being a dimension along which studies have been carried out, only three of the norming studies listed in Table 1 have measured this variable.

#### Familiarity

Familiarity is a fundamental index of conceptual knowledge, and in normative ratings for pictures and words it has been consistently correlated with measures including identification response latencies [25], [26], name agreement [1] and object categorization [4]. The importance of the familiarity effect has also been demonstrated in terms of differential brain response patterns (evoke related potentials) between familiar and unfamiliar sounds [27], [28]. Only two previous sound norming studies have looked at the relationship between familiarity and other measures: Marcell et al. [14] and Shafiro [26] reported that more familiar sounds were more accurately named. Two further studies reported mean familiarity ratings only [25], [29].

#### Confidence

How confident a person feels in their identification [14], [29] or categorization [29], [47] of a sound has previously been measured in sound norming studies (see Table 1). Although Schneider et al. [29] did not discuss their measures of confidence, their data showed a clear difference in confidence for participants naming and categorizing pictures compared with sounds. Marcell et al. [14] found a high correlation between confidence in naming and familiarity of a sound, leading them to suggest that these two measures index the same characteristic. The data collected here will allow us to verify this proposal.

#### Representativeness

How prototypical a sound is of the concept one is trying to represent is clearly fundamental to the experimentalist, and has been shown to have a significant effect on response time for both visual objects [30] and words [31]. Despite this, there have been no measures made of representativeness in the sound studies listed in Table 1. The nature of this measure is such that we would expect representativeness ratings to correlate strongly with both identification and familiarity, as the more representative a sound is of a concept, the easier we would expect it to be identified which would correspondingly relate to how familiar an item is.

#### Affect

Affective responses to acoustic stimuli have shown similar patterns of psychological and physiological responses to pictures. Specifically, the affective response to a target item can elicit physiological changes that reliably modulate responses in somatic, visceral and central systems, and this can subsequently impact on behavioral responses [32]. Interestingly, there are conflicting findings in the current literature on the influence of emotion. For example, Marcell et al. [14] measured pleasantness ratings for sounds and found that it did not influence naming accuracy, correlated only marginally with familiarity, and not at all with complexity. This led them to conclude that emotion is a relatively independent dimension. In contrast, Schneider et al [29] found that pleasantness ratings were highly correlated with familiarity of sounds. They interpreted this finding in the context of the mere exposure effect [33], where more familiar stimuli are usually perceived more positively than unfamiliar stimuli. Gaining a clearer understanding of this modulation of response is of particular importance for sound experiments investigating psychophysiological interactions, as well as investigations of how different affective dimensions impact on cognitive processes using paradigms such as evaluative conditioning or affective priming.

#### Imageability

For word and picture processing, the greater the imageability of a concept, the more likely that object is to be remembered and identified [34], [35]. There are currently no normative data available on the imageability of sounds, therefore this measure will provide novel information for understanding the role of imageability in sound recognition and identification.

### The Present Study

Given the limited number of studies currently available that provide comprehensive normative data for environmental sounds, our aim was to provide data on a large set of environmental sound stimuli designed for researchers investigating auditory processing across cognitive (neuro)psychological, psychophysiological and cognitive neuroscience domains. We obtained response time and identification data for a set of 110 common objects, natural events, human actions and animals, along with a comprehensive set of ratings of object categorization, familiarity, pleasantness, arousal and imageability. All environmental sounds have been made freely available online, along with a detailed table of the response data to assist researchers in the selection of the most suitable auditory objects for their own parameters of interest.

## General Materials and Methods

### Ethics Statement

The study complied with the Australian National Statement on Ethical Conduct in Human Research and was approved by The University of Queensland Medical Research Ethics Committee (Project #2007001910), including the process of providing consent online. In Study 1 participants provided their informed consent by clicking a checkbox prior to proceeding with the sound questionnaire. For Study 2, participants provided their written informed consent to the researcher prior to commencing.

### Selection of Sounds

Sounds had been collected as part of an ongoing database for stimuli used in fMRI studies by the first author [e.g., 36], primarily downloaded from the website www.sounddogs.com and www.freesounds.com. From this database, the same 110 natural sounds were used in Studies 1 and 2, with equal numbers of living and manmade items from 9 conceptual categories. All sounds were normalized to 1sec duration (16-bit 44,100Hz) using Audacity 1.2.5 (http://audacity.sourceforge.net) and presented in mp3 format in Study 1 (due to restrictions with the online software used) and wav format in Study 2. All files have been made available (in wav format) for download at http://www.imaging.org.au/Nessti. Also provided online are measures of concept frequency based on the Hyperspace Analogue to Language frequency norms (HAL) [39] for all available concepts, as well as the number of phonemes, syllables, and the Harmonics-to-Noise (HNR) Ratio.

## Study 1

The goal of Study 1 was to obtain a large set of normative data including naming, cognitive ratings and affective ratings, for 110 environmental sounds using an online questionnaire format. Due to the large number of sounds, the questionnaire was divided into 2 parts and participants could take part in either one or both of these questionnaires.

### Participants

There were 162 participants (85 male, 77 female), mean age 27.7 years (SD: 8.90), mean years of education 16 (SD: 3.27). See Table 2 for a summary of the participant demographics. One group of questionnaire respondents (73) were undergraduate students from the University of Queensland School of Health and Rehabilitation Sciences who were given course credit for participation. The second group of respondents (89) was recruited through on-line advertising to staff and students across the University of Queensland. These participants were encouraged to complete either one or both questionnaires by being entered into a draw to win a Macintosh iPad (both questionnaires completed meant 2 entries into the draw). In total we obtained 123 datasets for questionnaire 1 and 123 datasets for questionnaire 2, with 84 people completing both questionnaires. Although 5 participants reported a history of hearing impairment, their accuracy did not significantly differ from the remaining participants (p = .68).

**Table 2:** Demographic details for all respondents.

Demographic	Study 1	Study 2
Participants	162	53
Male	85	19
Female	77	34
Age mean (SD)	27.7 (8.9)	31.3 (10.84)
Education mean (SD)	16 (3.3)	16.47 (2.89)
Right Handed	145	49
Left Handed	15	3
Ambidextrous	1	1
*History of hearing impairment	6	2
History of mental illness	5	2
Australian citizen	119	30
Native language English	136	40

* Response accuracy for those participants reporting history of a hearing impairment did not differ from the remaining participants: Study 1: *t*[160] = −0.41, p = .68; Study 2: *t*[51] = 0.36, p = .72.

### Materials and Methods

An online questionnaire was developed and administered using the platform Questchain (www.questchain.com). Participants were given a link to the Internet page on which either part 1 or part 2 of the questionnaire could be accessed. The format of the self-paced questionnaire was as follows: Page 1 consisted of an information and consent form, which required the participant to give their informed consent to take part before being able to move to the next page. Page 2 described the study and gave the participant detailed instructions on what was required of them. At this point, the respondent was able to play a test sound to ensure the volume was set appropriately. Page 3 requested demographic and other personal information. The actual normative questionnaire started on the page 4, with one page per sound. The participant would first play the sound until they decided on their response, and then answer a series of questions. All questions had to be answered in order to advance to the next page. A screenshot of the page used to probe the participants’ knowledge regarding environmental sounds has been included in Supporting Information (Questionnaire S1).

The normative data collected were as follows:

#### Identification

The respondent was required to type the name of the sound.

#### Category

A list of categories was provided and the respondent was required to select one from the following nine options: Animal, Human, Nature, Household/Tool/Accessory, Recreational, Transport, Weapon, Alarm/Signal, Musical Instruments.

#### Familiarity rating

Participants were asked, “*How familiar is this sound?”.* Familiarity was rated using a 6-point scale [25] where 1 = highly familiar and 6 = highly unfamiliar. The respondent moved an icon with their computer mouse to indicate their choice of rating for this and the subsequent ratings.

#### Representativeness

Participants were asked, “*How representative/prototypical is this sound?*”. A 5-point scale was used where 1 = highly representative and 5 = highly unrepresentative.

#### Affective ratings

We utilized the Bradley and Lang [37] Self-Assessment Manikin (SAM) from the affective norms for English words (ANEW) for respondents to rate sounds on the affective dimensions of pleasure (happy vs. unhappy) and arousal (excited vs. calm) on a scale of 1 (happy/excited) –9 (unhappy/calm). For the pleasure dimension, participants were asked, “*Which face best describes how happy/unhappy you feel when you hear this sound?*”. For the arousal dimension, participants were asked, “*Which face best describes how excited/calm you feel when you hear this sound?*”.

### Data Analysis

#### Identification responses

To determine an agreed correctness for the identification response, three raters independently judged the accuracy of every participant response for each of the 110 concepts. This process created a set of synonyms for each environmental sound. These synonyms were then combined and a final list of acceptable responses was agreed upon. A summary of the agreed scoring guidelines is given in Table S1. In addition to the sound label given by the experimenters, synonyms included a description of the sound e.g., *caw* for the sound of a *crow*, an action associated with the sound e.g., *bounce* for the sound of a *ball*, all grammatically related forms of words e.g., bounce, bounced, bouncing, and basic level names for subordinate items e.g., bird vs. crow. Obvious misspellings were corrected. When the response to an item contained more than one word, a response was judged to be identical if the same words were used but in a different order e.g., the response “*dog barking*” was equivalent to “*barking dog*”.

A response was judged incorrect if:

1. A person did not use the target name or an agreed upon synonym in their response.
2. A description was given but without the target name or synonym e.g., *paper rustling* for the sound of scissors cutting paper, where scissors was the target name.
3. The response was a superordinate name e.g., animal for crow.
4. A number of alternative names were given (as we judged them to be guessing) e.g., the response *bird/mouse/monkey*, even if the target name was included in the list.
5. There was either no legible response or it was given as unknown.

For every environmental sound, we recorded the identification response and percent accuracy for each sound based on the above criteria. We also calculated what percentage of the identification responses were the same i.e., the modal response. For example, the number of correct responses for identifying the “cat” sound might be 100%, but the modal response may only have come from 92% of the respondents, with the remaining 8% being synonyms for the cat sound (e.g., feline, meow or kitten). We also provided a measure of name agreement using the *H*-value (see below). For categorization, we determined the modal category selected and the percentage correct category selected for each sound.

#### H-Value

The H-value is used to measure name agreement across respondents, and is indexed by the number of different names given to the same sound [4], [38]. It is calculated using the formula
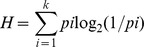
where *k* is the number of alternative names and *p_i_* is the proportion of subjects providing each of the alternative names. The value of *H* increases as a function of the number of alternative responses. Where an object receives total name agreement across all respondents, *H* = 0. The *H*-value was calculated based on correct responses in Studies 1 and 2 independently.

Correlational analyses assessed the relationship between mean ratings for familiarity, representativeness, pleasantness, arousal and identification (percent correct). Independent *t*-tests were also computed to assess any difference between living versus manmade sounds for cognitive and affective ratings or identification rate. Data analyses were carried out using IBM SPSS statistics v19.

## Results

### Identification

**Figure 1:**
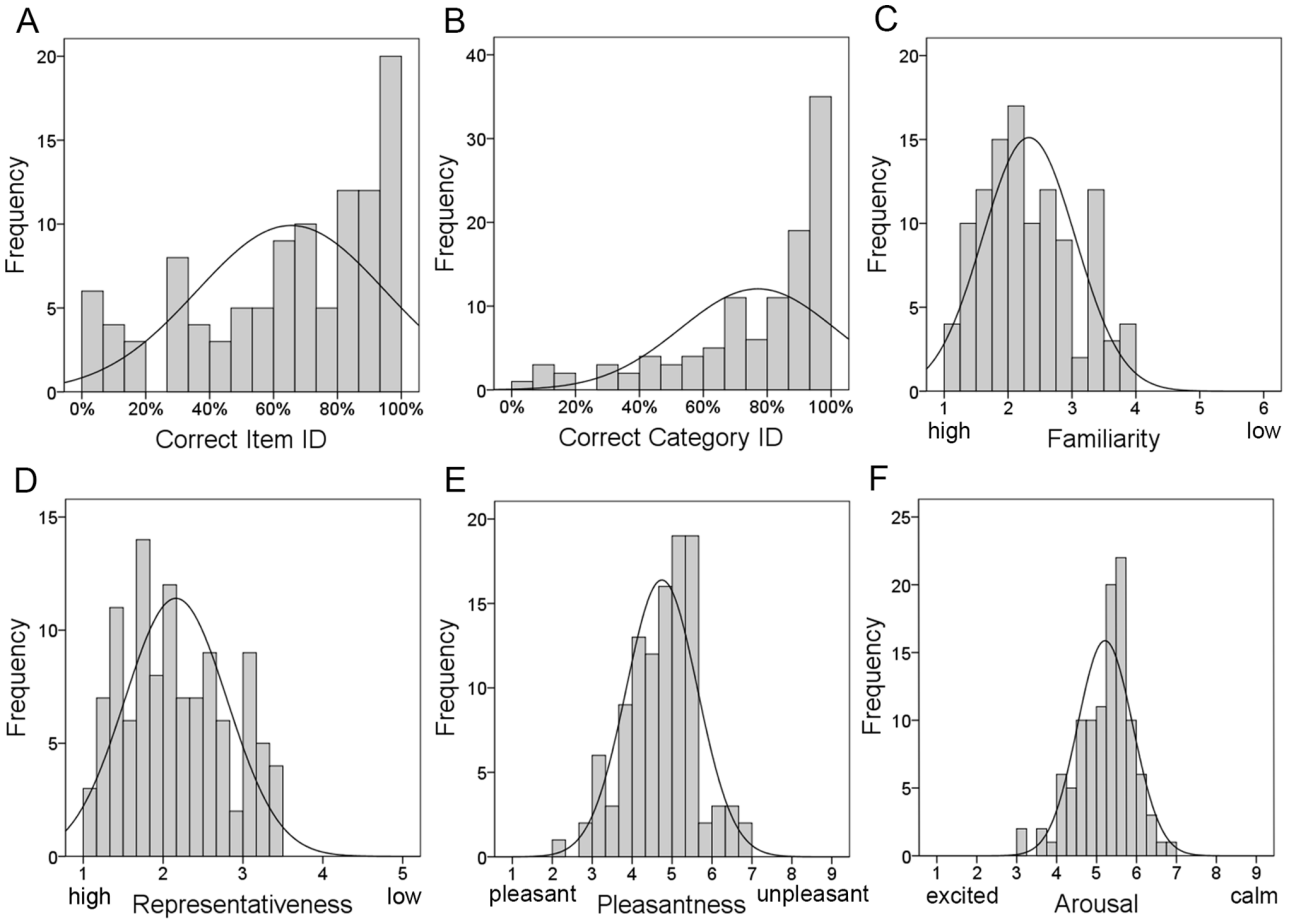
Frequency distributions for variables measured. Histograms show distribution frequencies and curves of best fit for: A) Correct item identification, B) Correct category identification, C) Familiarity ratings, where the higher the number the lower the familiarity, D) Representativeness, where the higher the number the less representative of an object the sound is, E) Affective ratings for pleasantness, where the higher the number the less pleasant the reaction is to the sound, and F) Arousal ratings, where the higher the number the more calm/sleepy the reaction is to the sound.

In Study 1, we received a total of 13515 correct responses out of a maximum of 13530. The proportion for all sounds correctly identified is listed in Table S2 and the frequency distribution is shown in Figure 1A. Five items were identified correctly by all participants (*cat, horse, laugh, phone, sneeze*), and 19 items were identified with a success rate of over 95%. The percentage of the sounds identified accurately across all participants was 65.31%. This accuracy was not dependent on whether the items were from a living (70.5% correct) or manmade (60.1% correct) category (*t*[108] = −1.87, *p* = .065). The scoring criteria for a correct name included multiple responses (i.e., acceptable synonyms, see Table S1), which meant that it was possible for the modal name to differ from the concept name; for example the modal name for *grasshopper* was *insect*. The modal name for 20% of sounds did not correspond to the expected label given to each sound by the experimenters, so for those cases we have provided the alternative modal name in parentheses in Table S2. It is also of note that some modal names were verbs rather than nouns (e.g., *sweeping* was the most common response for *broom*). The corresponding percentage of responses for each modal name is also given. The *H*-value was also calculated as a measure of name agreement on correct responses for each item and ranged from 0–4.90 (Table S2).

**Figure 2:**
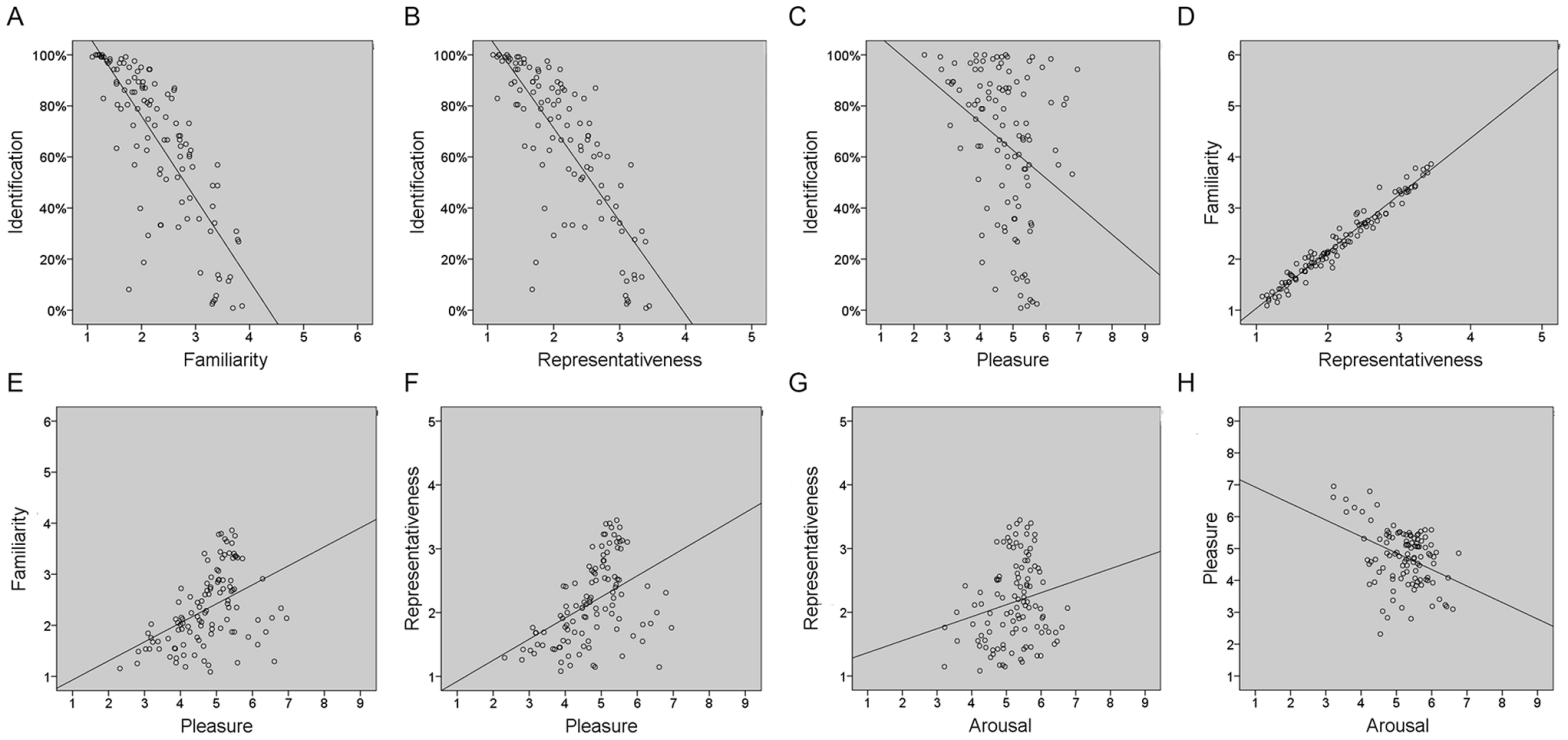
Scatterplots for significant correlations. Correlations with regression line are shown for: A) Identification/Familiarity, B) Identification/Representativeness, C) Identification/Pleasantness, D) Familiarity/Representativeness, E) Familiarity/Pleasantness, F) Representativeness/Pleasantness, G) Representativeness/Arousal, and H) Pleasantness/Arousal.

Level of identification was highly correlated with the familiarity rating for that item (*r*[110] = −.796, *p*<.0005), where the more likely a response was correct, the more familiar an item was rated to be. The measure of representativeness of a sound correlated with identification rate (*r*[110] = −.795, *p*<.0005) as did the rating of pleasantness (*r*[110] = −.335, *p*<.0005). There was no correlation between identification and arousal (*r*[110] = −0.143, *p* = .068) or frequency (*r*[110] = 0.046, *p* = .326). Figure 2A–C shows scatterplots for the significant correlations.

### Cognitive Ratings

On a scale of 1 - 6, where 1 = most familiar, mean familiarity rating ranged from 1.09–3.86 (M:2.33, SD:0.73). *Sneeze* was rated the most familiar and *skiing* was least familiar. There was a significant difference between the familiarity of living and manmade items (*t*[108] = 3.04, p = 0.003) with living things rated as more familiar than manmade sounds (see Table 3). Familiarity was highly correlated with representativeness (*r*[110] = .981, *p*<.0005). The high r-value suggests that these two ratings are indexing the same cognitive measure (Figure 2D). Familiarity also correlated significantly with pleasantness (*r*[110] = .458, *p*<.0005), which appeared to reflect the fact that ratings tended to be narrowly distributed about the mean on both scales. There was no clear relationship between familiarity and arousal (*r*[110] = .149, *p* = .06). Items are listed in Table S3 and the frequency distribution for familiarity ratings is shown in Figure 1C.

**Table 3:** Cognitive and affective ratings for living versus manmade concepts.

Study 1						
Category	N	ID %	FamiliarityMean (SD)	RepresentativenessMean (SD)	PleasantnessMean (SD)	ArousalMean (SD)
**Living**	55	70.5	2.12 (0.67)	1.97 (0.56)	4.66 (0.84)	5.27 (0.66)
**Manmade**	55	60.1	2.53 (0.73)	2.35 (0.66)	4.83 (0.94)	5.17 (0.73)
**Study 2**						
**Category**	**N**	**RT Mean (SD)**	**Confidence rating** **Mean (SD)**	**Imageability rating** **Mean (SD)**		
**Living**	55	2170 (391)	5.58 (0.95)	6.76 (1.29)		
**Manmade**	55	2276 (405)	4.94 (1.05)	5.97 (1.37)		

Representativeness was measured on a scale of 1–5 where 1 = highly representative (M:2.16, SD:0.64), and ranged from a horse which was judged to be a highly representative sound with a score of 1.08 to the sound of someone skiing given 3.45. Living and manmade items significantly differed on their ratings (t[108] = 3.18, p = .002), with living things judged to be more representative than manmade sounds (see Table 3). Ratings are listed in Table S3 and the frequency distribution is shown in Figure 1D. Unexpectedly, the rating for how representative a sound was of its source correlated with the rating for arousal (*r*[110] = .203, *p* = .017). The more representative an item was, the higher the rating of calmness (see Figure 2G for scatterplot).

### Affective Ratings

Ratings for pleasure were on a scale of 1 (most pleasant) - 9 (most unpleasant) with a range of 2.32–6.95 (M:4.74, SD:0.89). The sound rated most pleasant was *laugh* and most unpleasant was *machine gun*. Pleasure negatively correlated with arousal (*r*[110] = −.401, *p*<.0005). Arousal rating ranged from 3.21 (*fire alarm*) –6.76 (*yawn*) where 1 = most excited and 9 = most calm. (M:5.22, SD:0.69). There was no significant difference between living and manmade items on either pleasantness (t[108] = 1.04, p = 0.3) or arousal (t[108] = −0.81, p = .42) (see Table 3). Affective ratings are listed in Table S3 and the frequency distribution can be seen in Figures 1E–F. A scatterplot of the relationship between pleasantness and arousal is provided in Figure 2H.

### Sound Categorization

Participants classified each sound as belonging to one of 9 categories. The modal category response is provided against each sound in Table S2, as well as the proportion of respondents who selected that category. Only 9 sounds were categorized differently from those categories agreed by the raters, of which 4 were from the *Recreational* category and were alternatively categorized as being from the *Household* category (*pinball machine, basketball, book and skiing*). These 4 items also had low identification rates (30.89%, 29.27%, 13.01%, 1.63% respectively). For the remaining 5 sounds, *whistle* and *fire truck* had high identification rates (96.75% and 80.49% respectively) and were classified by the experimenters as *Alarm* and *Transport* whereas respondents classified them as *Recreational* and *Alarm*. The remaining 3 (*cicada, rockfall, washing machine*) had correspondingly low rates of correct identification (30.89%, 12.2%, 3.25% respectively). Table S4 summarizes the responses by category.

## Study 2

The aim of Study 2 was to obtain response latencies for identification of the same set of 110 sounds used in Study 1 under laboratory conditions, along with ratings of naming confidence and imageability of these sounds. We expected confidence and imageability to positively correlate with identification accuracy.

### Participants

There were 58 participants in total (22 male, 36 female), mean age 31.72 years (SD: 10.9), mean years of education 16.82 (SD: 3.33). Participants were recruited through advertising to staff and students across the University of Queensland. No volunteers who had participated in Study 1 took part in Study 2. 5 participants were excluded from the analysis due to technical difficulties with the recording equipment. Although 2 participants reported a history of hearing impairment, their accuracy did not significantly differ from the remaining participants (p = .72). See Table 2 for a summary of demographics.

### Procedure

This computer-based experiment was programmed in Cogent (www.ucl.ac.uk/vislab) and implemented in Matlab (Mathworks, Sherborne, MA, USA). Before beginning the experiment, participants were told that they would be hearing a large number of environmental sounds and their task was to verbally identify the object, animal or action depicted by the sound as quickly and clearly as possible. They would then be asked to rate how confident they were that their response was correct, and how imageable the sound was. Participants were asked not to describe or mimic the sound and were encouraged to use a single word response only. They were also asked not to use an article before the noun e.g. responding “dog” not “a dog”, or “ball” not “the ball”. A microphone was used for recording responses and reaction times, and sounds were played through headphones at each participant’s preferred volume. A brief practice session was run (using a different set of 6 sounds) and was repeated when necessary, with feedback from the experimenter, to correct any deviation from these instructions. The sound threshold for recording was adjusted for each individual during the practice trials in order to maximise recording sensitivity to their voice and minimise sensitivity to other extraneous noise. The time allowed to make a response was five seconds from the onset of the sound. After the 5 seconds, participants were then cued to provide their rating of 1) confidence in their response, and 2) the imageability of the sound. The normative data collected were as follows:

#### Identification

The respondent was required to name the object, animal or action that was making the sound.

#### Reaction time

This was measured from the onset of the environmental sound to the onset of the verbal response. Only correct responses were included in this analysis.

#### Confidence

Confidence was rated using a 7-point scale, where 1 = not confident and 7 = very confident. The participant had to answer “How confident are you in your decision?” by pressing the corresponding number on the keyboard.

#### Imageability

Imageability was rated using a 9-point scale [25] where 1 = no imagery and 9 = high imagery. The participant had to indicate how easily the sound brought an image to mind by pressing the corresponding number on the keyboard.

The same participants also carried out the same task on a corresponding set of pictures to identify and provide ratings for, but these data are not reported here. The sound experiment commenced prior to the picture experiment.

### Data analysis

#### Response latencies

Response latencies were manually extracted by 2 of the experimenters. One wav format sound file was created by Cogent for the participant’s response to each individual sound, with the onset of the recorded response file corresponding to onset of the sound stimulus. Response times were extracted from this track using Audacity. A standardized procedure for identifying the start point of the response involved amplifying the sound file using Audacity in order to manually visualize the start of the waveform, and zooming in to allow changes to be seen in the order of milliseconds. This manual method ensured that response times were not confounded by sounds other than the actual participant answer, which may have been detected had we used an automated procedure. This accuracy was verified by cross-checking with response latencies using an automated Matlab protocol. A random sample of reaction times were compared between experimenters to ensure inter rater-reliability.

Mean response latencies were computed for correct answers only. Correlational analyses assessed the relationships between mean response latency, correct identification percentage, mean confidence and mean imageability ratings. Independent *t*-tests analyzed the difference between living versus manmade sounds for identification percentage, reaction time, confidence and imageability. As for Study 1, all statistical analyses were computed using IBM SPSS statistics v19.

#### Rating responses

Accuracy of responses followed the same protocol as for Study 1 with three independent raters, but with changes to what was considered an acceptable response based on the different probe question (Study1: *Name the sound*; Study 2: *Name the object, animal or action*). The same set of noun synonyms was used for each environmental sound, including action words, grammatically related forms of words and basic level names for subordinate items. However for Study 2, a description of the sound was not considered correct (e.g., *caw* for the sound of a *crow*). The agreed scoring guidelines are provided in Table S1. Correlational analyses were then computed to evaluate the relationship between percent correct identification, mean response latency and mean confidence and imageability ratings. An Independent Samples *t*-test compared successful identification between Studies 1 and 2.

## Results

### Identification

**Figure 3:**
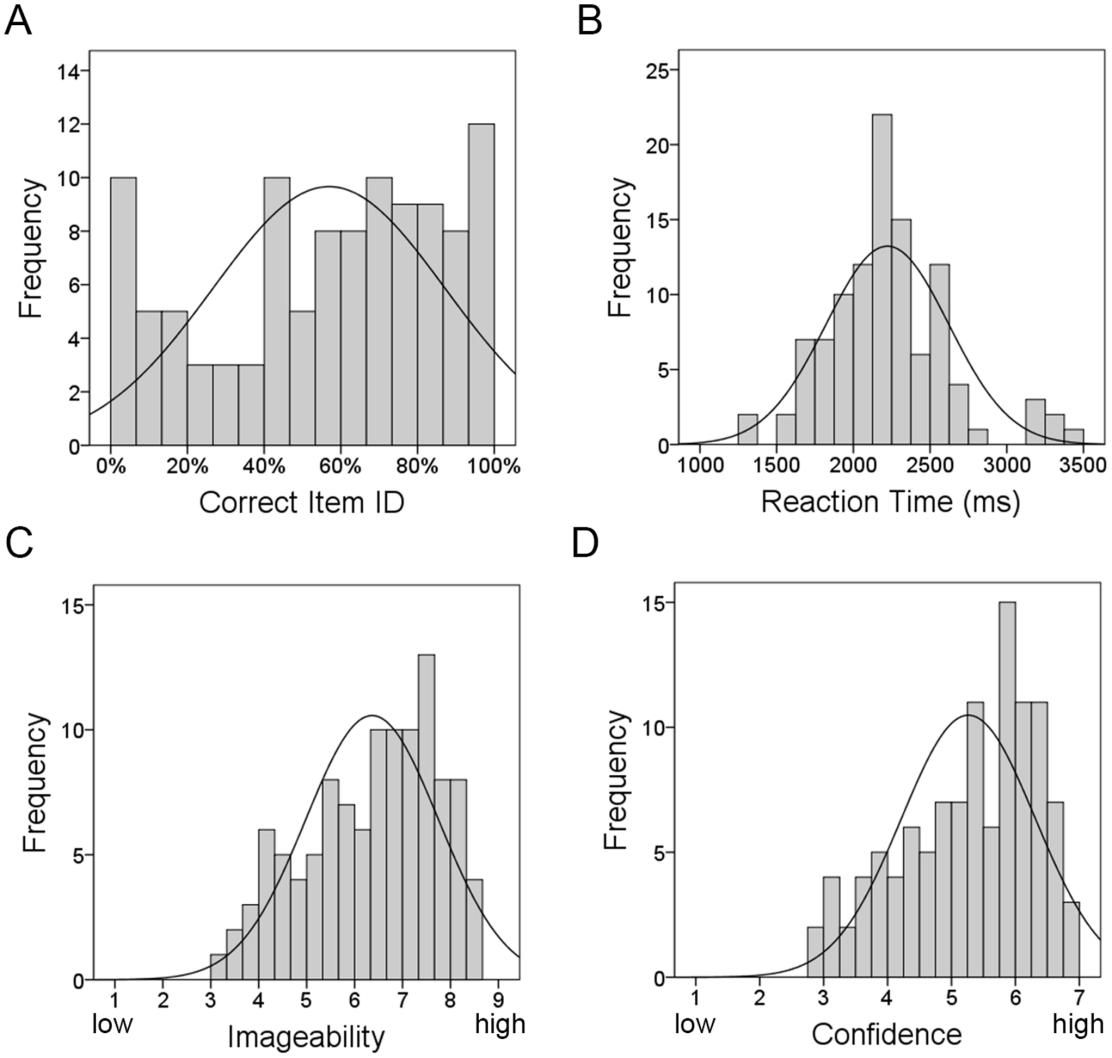
Frequency distributions for variables measured. Histograms show distribution frequencies and curves of best fit for: A) Correct item identification, B) Mean reaction time, C) Confidence ratings, where the higher the number the higher the confidence, D) Imageability ratings, where the higher the number the more imageable an object is.

**Figure 4:**
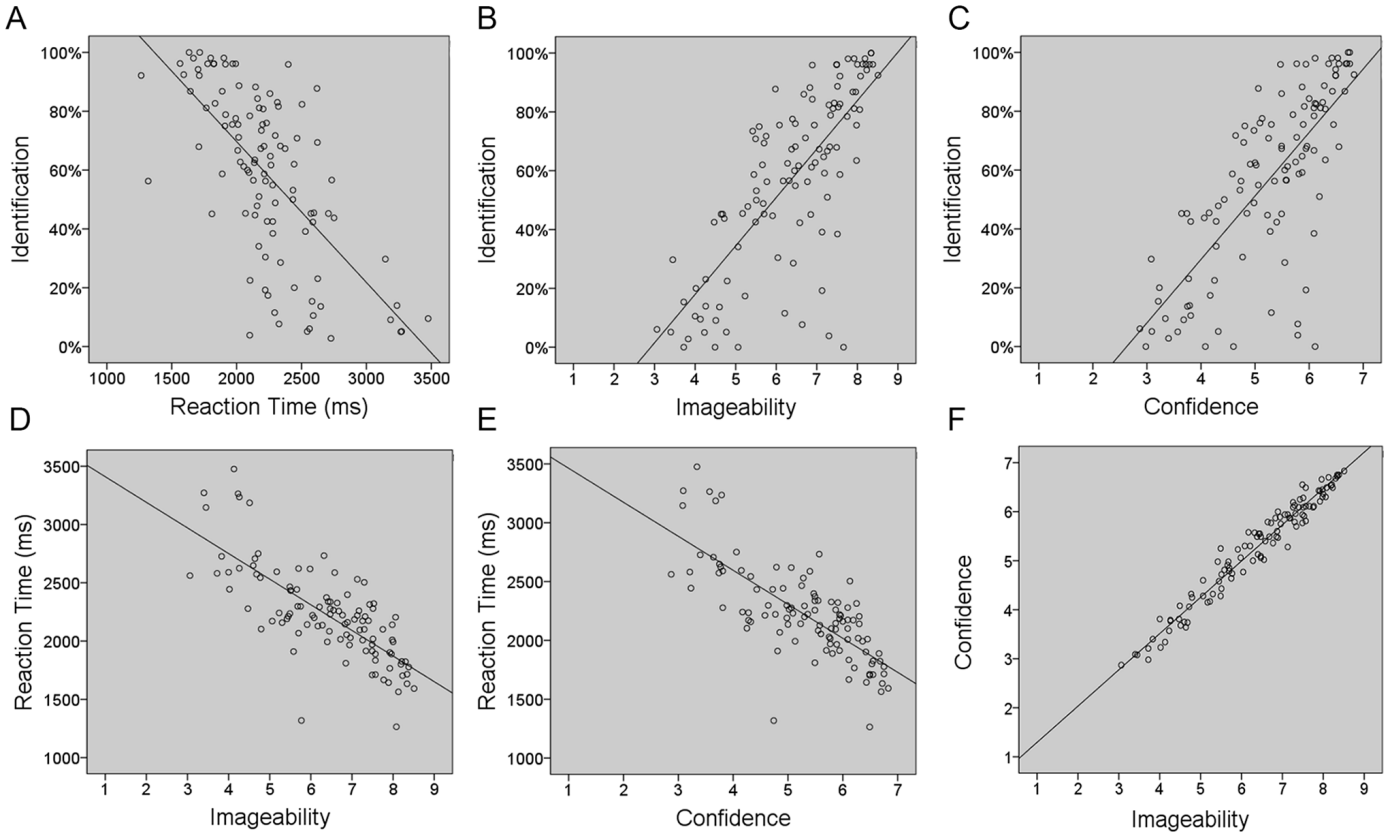
Scatterplots for significant correlations. Correlations with regression line are shown for: A) Identification/Reaction time, B) Identification/Imageability, C) Identification/Confidence, D) Reaction time/Imageability, E) Reaction time/Confidence, F) Confidence/Imageability.

In Study 2, there were a total of 5272 correct responses, from a maximum of 5830. Compared to Study 1 only two sounds were identified 100% correctly (*dog*, *horse*) and 13 sounds were identified with a success rate of over 95%. Across all participants, the mean percentage of sounds accurately identified was 56.91%. The proportion for all sounds correctly identified is listed in Table S5 and the frequency distribution is shown in Figure 3A. The mean identification percentage for manmade sounds was 51.68%, compared to 62.14% for living sounds, but this difference was not significant (*t*[108] = −1.83, *p* = .07). Identification highly correlated with mean reaction times (*r*[110] = −.67, *p*<.0005), where the faster a response, the more likely it was identified correctly. Identification correlated with ratings of confidence (*r*[110] = .75, *p*<.0005) and imageability (*r*[110] = .75, *p*<.0005). See Figures 4A–C for scatterplots of correlations.

### Reaction Time

Mean reaction times (RT) ranged from 1264–3476 msec (M: 2222, SD:400). *Sneeze* was identified the fastest (1264 msec), and *rock fall* required the longest time to be identified (3476 msec). RT also correlated with confidence (*r*[110] = −.75, *p*<.0005) and imageability (*r*[110] = −.75, *p*<.0005). See Figures 4D–E. RTs for manmade and living sounds were not significantly different (*t*[104] = 1.37, *p* = .17) (see Table 3). The frequency distribution for reaction time is shown in Figure 4B.

### Confidence and Imageability Ratings

Ratings for confidence were on a scale of 1 (low confidence) - 7 (high confidence) with a range of 2.87–6.83 (M: 5.26, SD: 1.05). The sound identified with most confidence was *cat* and with least confidence was *book*. The mean confidence ratings for manmade sounds versus living sounds was significantly different (*t*[108] = −3.34, *p* = .001), with participants more confident at identifying living sounds. Ratings for imageability were on a scale of 1 (no imagery) –9 (high imagery). Mean imageability of the sounds was 6.36 (SD: 1.38), and the concepts reflected those for confidence: *cat* was rated the highest for imageability (8.51) and sound of a *book* the least imageable (3.06). This was reflected in the high correlation between confidence and imageability ratings (*r*[110] = .98, *p*<.0005), see Figure 4F. The mean imageability ratings for manmade versus living sounds were significantly different (*t*[108] = −3.09, *p* = .003), with living things rated as more highly imageable than manmade sounds. Living versus manmade ratings are provided in Table 3 and the frequency distributions are shown in Figures 3C–D.

### Identification between Studies

An independent t-test was computed to evaluate if identification accuracy differed between the two types of studies. This showed a significant difference between studies, where participants were more accurate on the questionnaire in Study 1 compared to the lab-based design for Study 2 (*t*[218] = −2.08, *p* = .04). This result should be viewed with a note of caution however: although the sounds were the same, there was a difference in the number of times the sound could be heard between the two studies, as well as the type of identification question that was asked.

### Modal Names within and between Measures

The scoring criteria for a correct name included multiple responses (i.e., acceptable synonyms, see Table S1 & Table S5, for Study 1 and Study 2, respectively). This meant that it was possible for the modal name to differ from the target name, for example the modal name for *grasshopper* was *insect*. In Study 1, 20% and in Study 2, 31%, of the modal names for sounds did not correspond to the expected label given to each sound by the experimenters, so for those cases we have provided the alternative modal name in parentheses in Table S2 & Table S5. The corresponding percentage of responses for each modal name is also provided.

## General Discussion

The influence of variables such as frequency, familiarity or representativeness on recognition and identification have been well established for visual word and picture processing, yet the literature for environmental sounds is sparse. Our prime motivation for this study was to provide comprehensive normative data on a range of real living and manmade sounds of equivalent length, suitable for use across a wide range of empirical domains. All sound files have been made freely available online, including summaries of the variables for each item. We have also included commonly used lexical measures of word length, number of phonemes, number of syllables and frequency where available from the HAL frequency norms [36] to assist in stimulus selection should these sounds be utilized in conjunction with verbal auditory or visual stimuli.

The sounds used in this study covered a wide range of living and manmade concepts and a large set of cognitive and affective variables. Three previous studies have also measured a wide range of variables for the same sounds, but we found them inadequate in one or more areas when looking to use well-characterized sound stimuli in our own memory and language experiments. The most comprehensive data was obtained by Marcell et al. [14], but the variable sound length of the stimuli is inappropriate for use in studies where input duration needs to be equated, such as functional MRI studies using auditory objects. For example, processing a brief (<.15 sec) sound has been shown to elicit a differential and nonlinearly related BOLD response compared with a sounds of >6 sec duration [40]. Ballas [25] also provided a wide range of cognitive and affective measures, and equated their sound duration. However this was using a restricted set of 41 items consisting predominantly of manmade items. Finally, Schneider et al. [29] equated duration, tested multiple variables, and even obtained data for the pictures that corresponded to the sounds. However, they did report low identification rates for their sounds (see below).

The rate of correct environmental sound identification significantly differed between the two studies, which may be due to the participants only being able to hear the sound once in Study 2. It is also the case that the questions were framed differently for the two studies, and therefore the marking criteria were different because of this. Our identification rates are comparable to those of Ballas [25] (55%), who also used short sounds (0.625 sec). Interestingly the rate of identification reported here is highly favorable compared to that reported by Schneider et al. [29], who also used short sounds, and reported that identification of their sound stimuli was only 25% (compared with 84% accuracy for the corresponding pictures). We suggest that this low identification rate was due to the use of synthesized sound effects rather than natural sounds, as well as the short stimulus duration. Identification is clearly influenced by the length of the sound though, as studies using longer sounds have reported much higher identification rates. For example, Saygin, Dick, and Bates [41] reported a rate of 80.5% for a range of living and manmade sounds, and Marcell et al. [14] a rate of 80.97%. The development of sound norms necessarily involves a trade-off between the use of varied sound lengths which has ecological validity and optimizes recognition accuracy and the use of uniform lengths which is critical for certain experimental procedures (such as functional neuroimaging as described above) but will reduce recognition accuracy for certain naturally longer sound events. Unfortunately, the selection of varied sound durations to adequately capture the stimuli has typically relied on subjective judgment (see [14]) suggesting the need for further research, including the systematic testing of multiple durations of a single sound event to identify optimal recognition thresholds. By norming a large number of stimuli with a standard duration, the present study has identified stimuli that have relatively high accuracy and a uniform duration.

With regard to the relationship between identification and familiarity, the high correlation (*r* = .796) we report here replicates previous studies. For example, of the four previous studies that measured ratings of familiarity, three found that the more familiar an object, the significantly higher the likelihood that it would be identified correctly [14], [25], [26]. The fourth study measured familiarity but did not report any correlational analyses [29]. The strong correlation between familiarity and representativeness that we found with our data (*r* = .981) suggests that these two variables are indexing the same cognitive characteristic. Presumably this reflects the idea that if a sound is not representative of a concept, it is more difficult to determine if the concept is a familiar one.

We included measurement of standardized affective measures as these can have a significant effect on both physiological and behavioral responses [32]. We found a negative correlation between arousal and pleasure, where the more pleasurable the sound, the less arousing it was. However both affective ratings had only low standard deviations about a narrow, normal distribution, suggesting that these sounds were predominantly considered to be neutral. Interestingly, Schneider et al. [29] found a high correlation between familiarity and pleasantness across visual and auditory stimuli, and also reported that auditory stimuli clustered more around a neutral rating compared with ratings of the same concepts presented visually. We used the same ratings for pleasantness and arousal as Bradley & Lang [32], but found that the range of ratings for our stimuli was narrower (pleasure: 1.48–7.8 compared with 2.32–6.95 here; arousal: 1.31–8.34 compared with 3.21–6.76 here). However, this is not a surprising result when considered in the context of the Bradley & Lang [32] study, which was designed specifically to elicit strong emotions in order to activate physiological changes in response to auditory stimuli. Indeed, the stimulus set we have provided here will make selection of a larger number of neutral stimuli and control of valence possible for any studies not interested in measuring affective properties. It is also relevant to note that the length of the sounds used here may have shaped detection of emotion by rendering the sounds more neutral when presented so briefly. Indeed, Marcell et al [14] found that sound length correlated with affect, such that the longer the sound, the more pleasantly it was rated. This leads us to suggest that if a sound is unpleasant, it is rated as such independent of duration.

There is an established role for the modulating factor of semantic category membership on recognition and identification of words and pictures [42], [43] but far less is known about the influence of category membership on environmental sound processing, despite the literature on category-specific deficits in the neuropsychological literature. In Studies 1 and 2, we found no difference between identifying manmade versus living sounds, and although living things were named faster in Study 2, this difference was not signficant. Differences along the living/nonliving dimension have been reported by Fabiani, Kazmerski, Cycowicz, and Friedman [44], who found that across a range of different age groups, sounds from the human and animal categories were identified more easily. In an evaluation of category-specific differences in environmental sound processing, Giordano et al. [20] measured identification and response times to 140 sounds (71 living and 69 nonliving). They found that living sounds were identified more quickly and accurately than nonliving sounds. In a second experiment using a subset of 80 sounds, they revealed that participants focused more on symbolic mental representations for identification of living things and acoustic properties for nonliving things. Interestingly, we did find a difference between living and manmade sounds for cognitive ratings. Sounds of living things were rated to be significantly more familiar, representative and imageable. Participants were also more confident in their response to living compared with manmade sounds.

The findings for organizing living and manmade sounds along different dimensions reported by Giordano et al. [20] are of particular interest when considering the more traditional and predominant linguistic-based taxonomy for conceptual categories. Using this categorical taxonomy ensures ease of comparison across items such as pictures and sounds, but it is important to note that by continuing to use categories defined along visual or linguistic associations, we may be missing the ability to detect whether sounds are cognitively or neuroanatomically organized in a fundamentally different way. For example, in contrast to pictures, sounds may be organized by the context in which they are heard, rather than the objects that they are most closely related to. A relevant category of sounds could be that of “suburban environment” - a dog barking, a lawnmower running, and the sound of children playing. How do we then compare this type of category to the more traditional animal, tool and human categories? In one attempt to address this, Marcell et al. [14] asked their subjects for self-generated classifications. From 120 sounds, 27 category labels were generated. Interestingly, while some different categories did emerge from this process, those self-generated categories with a large number of members often resembled categories traditionally used and those employed in the present study (e.g., Signal; Animal; Human; Tool; Musical Instrument; Weapon) suggesting that commonalities exist regarding mental categorization across modalities. Further, while potential differences may exist in this categorical structure for sounds, it is not necessarily useful to researchers when comparing items across modalities such as pictures versus sounds or sounds versus words to redefine categories at this point. We therefore have remained with the more traditional living/manmade taxonomy, as well as providing some information on sub-categories, but with the caveat that this is not an entirely satisfactory distinction and further research on this topic at both behavioral and neurophysiological levels would be invaluable.

### Future Research and Conclusions

There are many aspects of sound processing that have not been the focus of the present study, but would be fruitful in enhancing our understanding of the different ways in which sounds are perceived, encoded and processed. These include the many low-level acoustic variables that are known to modulate behavioral perception as well as neuronal responses (e.g., [45], [46], [49]). Collecting the same data from older adults may also provide useful neuropsychological control data for clinical populations where naming and recognition deficits occur, which may also necessitate investigating how longer versions of these environmental sounds influence identification and ratings, as suggested by the effect for length reported by Marcell et al. [14]. The different ways in which objects are categorized would also provide valuable information, and this would be particularly relevant cross-culturally.

In summary, our aim was to provide a comprehensive set of ratings for natural living and manmade sounds that are also equated by a length suitable for use in a wide range of behavioral and neuroimaging settings. We have made these sounds freely available, and have provided a table of responses in order that the researcher has as much assistance in selection of appropriate and relevant stimuli as possible. Although there are many dimensions of environmental sound processing that still require attention, we hope that the data collected for these 110 unique sounds provides a level of description that will assist the researcher investigating environmental sound processing at multiple levels and in multiple research domains.

## Supporting Information

Table S1 **Scoring guidelines for judging naming responses in Studies 1 and 2.**
(DOCX)

Table S2 **Sound identification data and related response measures for each sound in Study 1.**
(DOCX)

Table S3 **Cognitive and affective rating variables for sounds in Study 1.**
(DOCX)

Table S4 **Classification and variable ratings by category for sounds in Study 1.**
(DOCX)

Table S5 **Sound identification data and related response measures for each sound in Study 2.**
(DOCX)

Questionnaire S1 **A screenshot of the online questionnaire used to probe the participants’ knowledge for each environmental sound.**
(DOCX)
