# Supplementary material for: NESSTI: Norms for Environmental Sound Stimuli
Source: PLoS One. 2013 Sep 4;8(9):e73382. doi: 10.1371/journal.pone.0073382 (PMC3762767; doi:10.1371/journal.pone.0073382)
Supplement: Table S1 — Scoring guidelines for judging naming responses in Studies 1 and 2. (DOCX) [file pone.0073382.s001.docx]

**Supporting Information**

**Table S1. Scoring guidelines for naming responses**

|  | **Description of the criteria for judging a correct response** | |
| --- | --- | --- |
| **Name** | **Sound Questionnaire** | **Sound Experiment** |
| Baby crying | The item root or synonyms e.g., *infant*, *toddler*. Related action e.g., *cry, wail.* | Same. |
| Basketball | The root *basketball* or *ball.* May be paired with root for actions *bounce* or *dribble*. | Same. |
| Bat | The root *bat.* Descriptors of the action *fly, wings* but not if bird also mentioned. | Same. |
| Bear | The root *bear* or *growl*. | The root *bear*. |
| Bee | The root *bee* or basic level *insect*. Root for sound descriptor *buzz*. | The root *bee* or basic level *insect*. |
| Bicycle | The root *bicycle*, *bike* or *bell*. Root of descriptors *wheel* or the action *ride*. | The root *bicycle*, *bike* or *bell*. Root for action *ride*. |
| Book | The root *book* or *page* or synonyms e.g., *hardback*, *novel*. | Same. |
| Broom | The root *broom*, *brush*. Root for the action *sweep*, *clean*. | Same. |
| Budgie | The root *budgie* or basic level *bird*. Root for sound descriptors e.g., *cheep*, *chirp*. | The root *budgie* or basic level *bird*. |
| Bull | The root *bull*, *ox* or basic level *cattle*. The sound descriptor *bellow*. | The root *bull*, *ox* or basic level *cattle*. |
| Burp | The root *burp*, *belch*. | Same. |
| Canary | The root *canary* or basic level *bird*. Appropriate sound descriptors e.g., *cheep*, *chirp*. | The root *canary* or basic level *bird*. |
| Cannon | The root *cannon* or synonyms e.g., *mortar*, *howitzer* or basic level *artillery*. Root for sound descriptors e.g., *explosion*, *blast*. | The root *cannon* or synonyms e.g., *mortar*, *howitzer* or basic level *artillery*. |
| Car | The root *car* or synonyms e.g., *auto*, *motor*. Root for action *drive*. Root for sound descriptors *squeal*, *screech* or *wheels*. | The root *car* or synonyms e.g., *auto*, *motor*. Root for action *drive.* |
| Cat | The root *cat* or synonyms e.g., *kitten* or basic level *feline*. Descriptor *meow*. | The root *cat* or synonyms e.g., *kitten* or basic level *feline*. |
| Chair | The root *chair* or synonyms e.g., *stool*, *seat* or basic level *furniture.* Root for action words *scrape*, *drag*. | Same. |
| Chicken | The root *chicken* or synonyms e.g., *hen, chook* or basic level *bird* or *poultry*. Root for sound descriptor *cluck*. | The root *chicken* or synonyms e.g., *hen, chook* or basic level *bird* or *poultry*. |
| Cicada | The root *cicada* or basic level synonyms *insect* and *bug*. | Same. |
| Clearing throat | The root *clear* and *throat.* Root for sound descriptors e.g., *cough*, *hack*. | Same. |
| Clock | The root *clock* or synonyms e.g., *watch*, *time*. The sound descriptor *tick.* | The root *clock* or synonyms e.g., *watch*, *time*. |
| Coffee machine | The root *coffee machine*, or basic level synonyms e.g., *kitchen appliance, café*. Root for action *grind*. | Same. |
| Coin | The root for *coin* or synonyms e.g., *change, dollar*. | Same. |
| Computer | The root *computer* or synonyms e.g., *PC, laptop* or descriptors *program, terminal*. Root for sound descriptors *beep, dial*. | The root *computer* or synonyms e.g., *PC, laptop.* |
| Cow | The root *cow* or *heifer* or basic level *cattle.* Root for sound descriptor *moo*. | The root *cow* or *heifer* or basic level *cattle.* |
| Cricket | The root *cricket* or basic level *insect*. | Same. |
| Crow | The root *crow* or *raven* or basic level *bird*. Root for sound descriptor *caw*. | The root *crow* or *raven* or basic level *bird*. |
| Cutlery | The roots for *cutlery* or synonym *utensil*, or specific descriptions e.g., *knife fork*. Root for sound descriptor *clang*. | The roots for *cutlery* or synonym *utensil*, or specific descriptions e.g., *knife fork*. |
| Dog barking | The root *dog* or synonyms e.g., *puppy, pooc*h or root for basic level *canine*. | Same. |
| Donkey | The root *donkey* or synonyms e.g., *mule*, *ass*. Root for sound descriptor e.g., *hee-haw, bray*. | The root *donkey* or synonyms e.g., *mule*, *ass*. |
| Door | The root *door* or basic level *entrance.* Root for related action e.g., *close, shut*. | Same. |
| Doorknob | The root *knob* or *door* synonyms e.g., *handle, hinge*. Root for the action *to lock*. | Same. |
| Drill | The root *drill* or basic level *power tool*, *electric tool*. | Same. |
| Drum | The root *drum* or synonym *bongo* or the root for basic level *percussion instrument*. | Same. |
| Duck | The root *duck* or basic level *bird.* Root for sound descriptor *quack*. | The root *duck* or basic level *bird.* |
| Elephant | The root *elephant* or descriptor *trunk*. | Same. |
| Fire | The root *fire* or verb synonyms e.g., *flame, blaze*. Root for sound descriptor *crackling*. | The root *fire* or verb synonyms e.g., *flame, blaze*. |
| fire alarm | The roots *alarm*, *siren*, *fire*. Root for descriptors e.g., *alert*, *emergency*. | The roots *alarm*, *siren*, *fire*. |
| Fire truck | The root *fire truck*, *fire engine* or basic level *emergency vehicle.* Root for sound descriptors e.g., *siren, alarm*. | Same. |
| Flute | The root *flute* or synonyms *windpipe, piccolo* or basic level *wind* *instrument*. | Same. |
| Fly | The root *fly* or basic level synonyms e.g., *insect*, *bug.* Root for sound descriptor *buzz*. | The root *fly* or basic level synonyms e.g., *insect*, *bug.* |
| Footsteps | The root *foot*, *step* or synonyms e.g., *walk, tread*. Root for object e.g., *shoe, heel*. | Same. |
| Frog | The root *frog*, *toad* or basic level *reptile*. Root for sound descriptor e.g., *croaks*, *ribbit*. | The root *frog*, *toad* or basic level *reptile*. |
| Gargle | The root for *gargle* or descriptors *mouthwash* or *throat*. | Same. |
| Goat | The root *goat* or synonym *kid*. Root for sound descriptor *baa*. | The root *goat* or synonym *kid*. |
| Gong | The root *gong* or basic level *percussion* *instrument*. | Same. |
| Goose | The root *goose* or basic level *bird.* Root for sound descriptor *honk.* | The root *goose* or basic level *bird.* |
| Grasshopper | The root *grasshopper* or basic level *insect*, *bug*. | Same. |
| Guitar | The root *guitar*, *banjo* or basic level *string instrument*. Root for action *strum*. | Same. |
| Helicopter | The root *helicopter,* *chopper* or basic level *aircraft*. | Same. |
| Horse | The root *horse* or synonyms e.g., *stallion*. Root for sound *neigh*. | The root *horse* or synonyms e.g., *stallion*. |
| Keys | The root for *key* or verb *jingle*. | The root for *key.* |
| Knife | The root *knife*, *blade* or basic level *kitchen utensil*. Root for action synonyms e.g., *chop, cut.* | Same. |
| Kookaburra | The root *kookaburra* or basic level *bird* or the root for *laugh*. | The root *kookaburra* or basic level *bird.* |
| Laugh | The root *laugh* or synonyms e.g., *cackle, chuckle*. Root for descriptor *happy*. | The root *laugh* or synonyms e.g., *cackle, chuckle*. |
| Lighter | The root *lighter* or descriptors e.g., *match, smoke*. | The root *lighter.* |
| Lion | The root *lion* or synonyms e.g., *cub* or basic level *feline, big cat* or the root for sound descriptor *roar*. | The root *lion* or synonyms e.g., *cub* or basic level *feline, big cat.* |
| Machinegun | The root *machinegun* or synonyms e.g., *ouzi*. Root for *action shoot* and sound descriptors e.g., *rapid-fire.* | The root *machinegun* or synonyms e.g., *ouzi*. Root for *action shoot*. |
| Maracas | The root *maracas* or synonym *shakers* or basic level *percussion instrument*. | Same. |
| Match | The root *match* or descriptors e.g., *light, strike, flame*. | The root *match* or the actions *to light or strike.* |
| Microwave | The root *microwave* or basic level synonyms e.g., *kitchen appliance, oven*. | Same. |
| Monkey | The root *monkey*, *chimp, ape* or basic level *primate*. | Same. |
| Mosquito | The root *mosquito* or basic level *insect.* Root for sound descriptor *buzz*. | The root *mosquito* or basic level *insect.* |
| Mouse | The root *mouse* or basic level *rodent*. Root for sound descriptor *squeak*. | The root *mouse* or basic level *rodent*. |
| Music box | The root *music box* or synonyms e.g., *jewelry box, music toy*. | Same. |
| Noisy miner | The root *miner* or basic level *bird*. Root for sound descriptors e.g., *cheep, twitter*. | The root *miner* or basic level *bird*. |
| Ocean | The root *ocean* or synonyms e.g., *sea, beach, surf, wave*. | Same. |
| Owl | The root *owl* or basic level *bird.* Root for sound descriptors e.g., *hoot, tu-whit-tu-whoo*. | The root *owl* or basic level *bird.* |
| Paper | The root *paper* or synonyms *sheet*, *note, poster*. | Same. |
| Parrot | The root *parrot* or synonyms e.g., *parakeet, lorikeet* or basic level *bird*. Root for the sound descriptor *squawk*. | The root *parrot* or synonyms e.g., *parakeet, lorikeet* or basic level *bird.* |
| Pen | The root *pen*, *biro or marker*. Root for action descriptors e.g., *write, click.* | Same. |
| Phone | The root *phone* or synonyms e.g., *handset, mobile*. Root for descriptors e.g., *call, ring*. | The root *phone* or synonyms e.g., *handset, mobile*. |
| Piano | The root *piano* or synonym *keyboard* or basic level *musical instrument.* | Same. |
| Pig | The root *pig* or *hog.* Root for sound descriptors e.g., *snort, grunt*. | The root *pig* or *hog.* |
| Pigeon | The root *pigeon*, *dove* or basic level *bird.* Root for the sound descriptor *coo*. | The root *pigeon*, *dove* or basic level *bird.* |
| Pinball machine | The root *pinball* or basic level *game*. | Same. |
| Plane | The root or synonyms *aircraft* or *jet*. Root of verb *fly*. | Same. |
| Plates | The root *plate* or synonyms e.g., *dish*, *crockery* or basic level *kitchen utensils*. | Same. |
| Printer | The root *print* or synonyms e.g., *copier*, *laser* or basic level *technology.* Root for the verb *to copy*. | Same. |
| Radio | The root *radio* or *stereo* or object *man* or basic level *technology*. Root for radio sounds e.g., *talk, voice*. | The root *radio* or *stereo* or basic level *technology*. |
| Rain | The root *rain* or synonyms *pour, drizzle, storm* or *wet weather*. | Same. |
| River | The root *river* or synonyms e.g., *stream*, *brook* or basic level *water*. | Same. |
| Rock fall | The root *rock* or synonyms *avalanche*, *landslide* or sound descriptor *earthquake*. | Same. |
| Rooster | The root *rooster*, synonym *cock*, or basic level *bird.* Root for the sound descriptor *crow*. | The root *rooster*, synonym *cock*, or basic level *bird.* |
| Saxophone | The root *sax* or basic level *brass instrument.* Root for sound descriptor *jazz*. | Same. |
| Scissors | The root *scissor* or synonym *shears*, or the root for action synonyms e.g., *clip, cut*. | Same. |
| Seal | The root *seal* or synonym *sea lion* or basic level *sea mammal*. | Same. |
| Sheep | The root *sheep* or synonyms e.g., *lamb, ewe*. Root for the sound descriptor *baa*. | The root *sheep* or synonyms e.g., *lamb, ewe*. |
| Shower | The root *shower* or descriptors *bath*, *water*. |  |
| Skiing | The root *ski* or descriptors *snow*, *ice*. Root for the actions *skiing* or *sliding.* | Same. |
| Sneeze | The root *sneeze*, or descriptors e.g., *cold, flu, man*. Root for the descriptor *achoo*. | The root *sneeze*, or descriptors e.g., *cold, flu.* |
| Snore | The root *snore* or descriptors e.g., *sleep, snooze*. | Same. |
| Tambourine | The root *tambourine*, *pandeiro or* *timbrel* or basic level *percussion* *instrument*. | Same. |
| Tennis | The root *tennis* or the root for descriptors *ball*, *racquet*. Root for actions *bounce*, *hit*. | Same. |
| Toaster | The root *toaster* or basic level *kitchen appliance*. | Same. |
| Toilet | The root *toilet* or synonyms e.g., *WC, lavatory* or basic level *bathroom*. Root for the verb *to flush*. | Same. |
| Train | The root *train* or synonyms *locomotive*, *rail, subway* or *engine*. | Same. |
| Triangle | The root *triangle* or basic level *percussion instrument.* Root for the sound descriptors *ding* and *ting*. | The root *triangle* or basic level *percussion instrument.* |
| Trumpet | The root *trumpet* or basic level *brass instrument*. | Same. |
| Turkey | The root *turkey* or basic level *bird.* Root for sound descriptor *gobble*. | The root *turkey* or basic level *bird.* |
| Vacuum cleaner | The root *vacuum* or synonyms e.g., *hoover* or basic level *cleaning*. | Same. |
| Washing machine | The root *wash* or basic level *laundry*. | Same. |
| Water bubbling | The root *water* or the root for sound descriptor *bubble*. | Same. |
| Whale | The root *whale*, *orca, marine life* or basic level *sea mammal*. | Same. |
| Whip | The root *whip* or synonym *cane*, or the root for actions e.g., *thrash, beat*. | Same. |
| Whistle | The root *whistle* or object *referee*. | Same. |
| Whistling | The root *whistle*. | Same. |
| Wind | The root *wind* or synonyms e.g., *gale, breeze*, *storm* or the root for *air*. | Same. |
| Wind chime | The root *chime or wind.* | Same. |
| Wolf | The root *wolf* or basic level *wild dog, canine.* Root for the sound descriptor *howl*. | The root *wolf* or basic level *wild dog, canine.* |
| Yawn | The root *yawn.* The sound descriptors *sleepy* and *tired*. | The root *yawn.* |
