# Supplementary material for: NESSTI: Norms for Environmental Sound Stimuli
Source: PLoS One. 2013 Sep 4;8(9):e73382. doi: 10.1371/journal.pone.0073382 (PMC3762767; doi:10.1371/journal.pone.0073382)
Supplement: Table S2 — Sound identification data and related response measures for each sound in Study 1. (DOCX) [file pone.0073382.s002.docx]

**Supporting Information**

**Table S2. Sounds in Study 1 listed in order of identification accuracy with percentage correct, modal response, H-value and category classification with proportion correct**

| **Sound** | **Number of Correct Responses** | **ID % Correct** | **% Modal Response** | **H-Value** | **Modal Category** | **% Modal Response** |
| --- | --- | --- | --- | --- | --- | --- |
| Laugh | 123 | 100.00 | 100.00 | 0.00 | H | 99.19 |
| Horse | 123 | 100.00 | 95.93 | 1.54 | A | 99.19 |
| Sneeze | 122 | 100.00 | 94.26 | 0.08 | H | 97.56 |
| Cat | 123 | 100.00 | 91.87 | 1.71 | A | 99.19 |
| Phone | 123 | 100.00 | 65.85 | 3.59 | HTA | 80.49 |
| Helicopter | 122 | 99.19 | 98.36 | 1.37 | T | 99.19 |
| Piano | 122 | 99.19 | 92.62 | 1.78 | MI | 97.56 |
| Clearing throat | 122 | 99.19 | 85.25 | 0.85 | H | 95.93 |
| Dog barking | 122 | 99.19 | 59.02 | 1.06 | A | 99.19 |
| Car | 121 | 98.37 | 90.08 | 4.88 | T | 98.36 |
| Snore | 120 | 97.56 | 98.33 | 0.63 | H | 94.31 |
| Frog | 120 | 97.56 | 94.17 | 1.73 | A | 97.56 |
| Rooster | 120 | 97.56 | 88.33 | 1.92 | A | 100.00 |
| Chicken | 120 | 97.56 | 83.33 | 1.44 | A | 98.37 |
| Whistling | 119 | 97.54 | 100.00 | 0.37 | H | 93.50 |
| Whistle | 119 | 96.75 | 101.68 | 2.81 | R | 31.71 |
| Canary (MR: Bird) | 119 | 96.75 | 94.12 | 2.72 | A | 87.70 |
| Coin | 117 | 95.12 | 97.44 | 4.33 | HTA | 78.86 |
| Baby crying | 117 | 95.12 | 82.05 | 1.03 | H | 92.68 |
| Guitar | 116 | 94.31 | 99.14 | 2.03 | MI | 99.19 |
| Cow | 116 | 94.31 | 93.97 | 1.34 | A | 96.75 |
| Machine gun | 116 | 94.31 | 46.55 | 3.24 | W | 91.80 |
| Water bubbling | 116 | 94.31 | 43.97 | 4.68 | N | 66.67 |
| Gargle | 115 | 93.50 | 99.13 | 2.39 | H | 92.68 |
| Flute | 110 | 90.16 | 92.73 | 0.84 | MI | 98.36 |
| Drum | 110 | 89.43 | 95.45 | 2.57 | MI | 98.37 |
| Toilet | 110 | 89.43 | 92.73 | 0.96 | HTA | 91.06 |
| River (MR: Water running) | 110 | 89.43 | 33.64 | 4.90 | N | 94.31 |
| Budgie (MR: Bird) | 109 | 89.34 | 78.90 | 2.73 | A | 90.24 |
| Crow (MR: Bird) | 109 | 89.34 | 65.14 | 2.51 | A | 95.12 |
| Saxophone | 109 | 88.62 | 90.83 | 1.22 | MI | 99.19 |
| Owl | 108 | 87.80 | 91.67 | 1.74 | A | 91.87 |
| Parrot (MR: Bird) | 107 | 86.99 | 85.05 | 3.22 | A | 92.68 |
| Paper | 107 | 86.99 | 61.68 | 4.90 | HTA | 67.48 |
| Plane | 107 | 86.99 | 42.99 | 3.34 | T | 91.06 |
| Donkey | 106 | 86.18 | 99.06 | 1.00 | A | 91.06 |
| Trumpet | 106 | 86.18 | 98.11 | 1.19 | MI | 98.37 |
| Elephant | 105 | 85.37 | 100.00 | 0.65 | A | 86.18 |
| Yawn | 105 | 85.37 | 98.10 | 0.38 | H | 100.00 |
| Door | 104 | 84.55 | 102.88 | 3.85 | HTA | 86.89 |
| Bicycle (MR: Bell) | 102 | 82.93 | 67.65 | 3.77 | T | 57.38 |
| Fire alarm (MR: Siren) | 102 | 82.93 | 55.88 | 2.37 | AS | 83.74 |
| Duck | 101 | 82.11 | 68.32 | 2.27 | A | 96.75 |
| Bee | 100 | 81.30 | 92.00 | 2.25 | A | 80.49 |
| Wind chimes | 100 | 81.30 | 52.00 | 1.75 | MI | 52.85 |
| Sheep | 99 | 80.49 | 93.94 | 1.48 | A | 97.56 |
| Monkey | 99 | 80.49 | 91.92 | 1.37 | A | 98.37 |
| Fire truck (MR: Siren) | 99 | 80.49 | 60.61 | 3.62 | AS | 8.13 |
| Kookaburra | 97 | 79.51 | 76.29 | 2.07 | A | 95.93 |
| Cricket | 96 | 78.05 | 100.00 | 1.34 | A | 77.24 |
| Radio | 93 | 76.23 | 84.95 | 3.82 | HTA | 55.28 |
| Turkey | 92 | 74.80 | 72.83 | 2.13 | A | 95.93 |
| Tambourine | 91 | 73.98 | 93.41 | 1.00 | MI | 96.75 |
| Match | 90 | 73.17 | 94.44 | 2.17 | HTA | 78.69 |
| Music box | 89 | 72.36 | 79.78 | 2.57 | MI | 55.28 |
| Footsteps (MR: Walking) | 89 | 72.36 | 53.93 | 3.85 | H | 69.11 |
| Broom (MR: Sweeping) | 87 | 70.73 | 85.06 | 2.64 | HTA | 89.43 |
| Whip | 84 | 68.29 | 85.71 | 1.67 | W | 36.59 |
| Clock | 84 | 68.29 | 82.14 | 2.10 | HTA | 73.77 |
| Wolf | 83 | 67.48 | 85.54 | 1.56 | A | 100.00 |
| Lion | 82 | 66.67 | 87.80 | 2.10 | A | 93.50 |
| Fire | 82 | 66.67 | 81.71 | 2.46 | N | 71.54 |
| Goose | 82 | 66.67 | 73.17 | 2.15 | A | 97.54 |
| Pig | 80 | 65.04 | 97.50 | 1.59 | A | 82.11 |
| Maracas | 79 | 64.75 | 73.42 | 2.29 | MI | 82.11 |
| Gong | 79 | 64.23 | 93.67 | 1.62 | MI | 91.87 |
| Rain | 78 | 63.41 | 98.72 | 1.41 | N | 65.04 |
| Pigeon | 77 | 62.60 | 64.94 | 2.56 | A | 97.56 |
| Cannon (MR: Explosion) | 77 | 62.60 | 54.55 | 3.34 | W | 60.98 |
| Cutlery | 75 | 60.98 | 85.33 | 3.35 | HTA | 95.12 |
| Train | 74 | 60.16 | 90.54 | 2.38 | T | 61.79 |
| Pen | 74 | 60.16 | 79.73 | 3.88 | HTA | 69.11 |
| Chair | 70 | 57.38 | 84.29 | 4.25 | HTA | 78.05 |
| Fly | 70 | 57.38 | 77.14 | 0.18 | A | 85.37 |
| Noisy miner (MR: Bird) | 69 | 56.10 | 92.75 | 2.14 | A | 89.43 |
| Wind | 68 | 55.28 | 89.71 | 2.91 | N | 56.91 |
| Vacuum cleaner | 67 | 54.47 | 74.63 | 2.53 | HTA | 89.43 |
| Mosquito | 65 | 53.28 | 76.92 | 1.75 | A | 72.13 |
| Burp | 64 | 52.03 | 92.19 | 0.58 | H | 49.59 |
| Ocean (MR: Waves) | 63 | 51.22 | 71.43 | 3.58 | N | 79.67 |
| Seal | 60 | 49.59 | 95.00 | 1.38 | A | 87.80 |
| Computer | 60 | 48.78 | 60.00 | 3.63 | HTA | 45.53 |
| Keys | 53 | 43.09 | 98.11 | 2.56 | HTA | 90.24 |
| Microwave | 52 | 42.28 | 100.00 | 0.97 | HTA | 86.99 |
| Shower (MR: Water) | 50 | 40.65 | 56.00 | 4.15 | HTA | 82.64 |
| Triangle | 48 | 39.02 | 83.33 | 1.63 | MI | 51.22 |
| Doorknob (MR: Door) | 44 | 35.77 | 134.09 | 3.55 | HTA | 85.37 |
| Printer | 43 | 34.96 | 72.09 | 2.78 | HTA | 58.54 |
| Lighter | 42 | 34.15 | 88.10 | 3.08 | HTA | 84.43 |
| Mouse | 41 | 33.33 | 78.05 | 1.71 | A | 88.62 |
| Grasshopper (MR: Insect) | 40 | 32.52 | 92.50 | 1.91 | A | 60.98 |
| Pinball machine | 38 | 30.89 | 86.84 | 0.96 | HTA | 40.65 |
| Drill | 38 | 30.89 | 65.79 | 1.83 | HTA | 93.50 |
| Cicada (MR: Insect) | 38 | 30.89 | 63.16 | 2.17 | HTA | 31.97 |
| Basketball (MR: Ball bouncing) | 36 | 29.27 | 16.67 | 1.86 | HTA | 30.89 |
| Plates | 34 | 27.64 | 55.88 | 4.51 | HTA | 79.67 |
| Tennis | 28 | 22.76 | 92.86 | 4.20 | R | 38.21 |
| Goat | 23 | 18.70 | 113.04 | 1.21 | A | 99.19 |
| Knife (MR: Chopping) | 18 | 14.63 | 94.44 | 3.52 | HTA | 65.04 |
| Scissors (MR: Cutting) | 17 | 13.82 | 82.35 | 2.14 | HTA | 66.67 |
| Book | 16 | 13.01 | 81.25 | 3.69 | HTA | 11.38 |
| Rock fall (MR: Rocks) | 15 | 12.20 | 100.00 | 3.16 | HTA | 19.51 |
| Whale | 14 | 11.38 | 92.86 | 1.79 | A | 40.65 |
| Bull (MR: Cattle) | 10 | 8.13 | 60.00 | 2.05 | A | 98.37 |
| Bat | 7 | 5.69 | 28.57 | 1.84 | A | 72.36 |
| Coffee machine | 5 | 4.10 | 80.00 | 0.72 | HTA | 72.13 |
| Washing machine | 4 | 3.25 | 100.00 | 0.00 | T | 43.90 |
| Bear | 3 | 2.44 | 100.00 | 1.50 | A | 47.15 |
| Toaster | 1 | 0.82 | 100.00 | 0.00 | HTA | 70.73 |
| Skiing (MR: Ice) | 1 | 0.81 | 200.00 | 0.00 | HTA | 5.69 |

Data includes the target name of the sound, with modal response in parentheses where this did not match the target; percent correctly identified; percent of responses that matched the modal response (i.e. not synonyms); the H-value for name agreement; modal response for category of the target sound; percentage correct category judgments. Key: MR = Modal Response; AS = Alarm/Signal; A = Animal; H = Human; HTA = Household/Tool/Accessory; MI = Musical instruments; N = Nature; R = Recreational; T = Transport; W = Weapon.
