# Supplementary material for: NESSTI: Norms for Environmental Sound Stimuli
Source: PLoS One. 2013 Sep 4;8(9):e73382. doi: 10.1371/journal.pone.0073382 (PMC3762767; doi:10.1371/journal.pone.0073382)
Supplement: Table S3 — Cognitive and affective rating variables for sounds in Study 1. (DOCX) [file pone.0073382.s003.docx]

**Supporting Information**

**Table S3.** **Mean and standard deviation for responses to**

**cognitive and affective rating variables for Study 1 sounds**

| **Sound** | **Familiarity**  **Mean (SD)** | **Represent. Mean (SD)** | **Pleasantness**  **Mean (SD)** | **Arousal**  **Mean (SD)** |
| --- | --- | --- | --- | --- |
| Baby crying | 1.77 (0.96) | 1.63 (0.92) | 5.89 (1.94) | 4.28 (1.72) |
| Basketball | 2.12 (0.96) | 2.00 (0.88) | 4.06 (1.29) | 5.13 (1.80) |
| Bat | 3.38 (1.56) | 3.09 (1.19) | 5.23 (1.61) | 5.28 (1.64) |
| Bear | 3.31 (1.29) | 3.11 (1.02) | 5.72 (1.53) | 4.92 (1.83) |
| Bee | 2.11 (1.07) | 1.81 (0.88) | 6.15 (1.57) | 4.03 (1.69) |
| Bicycle | 2.56 (1.21) | 2.46 (1.01) | 4.27 (1.43) | 5.30 (1.58) |
| Book | 3.64 (1.31) | 3.33 (1.06) | 5.28 (1.19) | 5.60 (1.62) |
| Broom | 2.88 (1.21) | 2.40 (0.88) | 5.40 (1.12) | 5.41 (1.81) |
| Budgie | 2.24 (1.03) | 2.11 (0.96) | 4.30 (1.59) | 5.54 (1.70) |
| Bull | 1.76 (1.06) | 1.67 (0.91) | 4.46 (1.36) | 5.18 (1.62) |
| Burp | 2.67 (1.27) | 2.44 (1.10) | 5.41 (1.59) | 5.59 (1.54) |
| Canary | 1.67 (0.75) | 1.50 (0.65) | 3.23 (1.56) | 6.39 (1.80) |
| Cannon | 2.91 (1.45) | 2.41 (1.09) | 6.28 (1.69) | 3.81 (1.59) |
| Car | 1.62 (0.62) | 1.55 (0.60) | 6.15 (1.73) | 3.58 (1.60) |
| Cat | 1.22 (0.49) | 1.17 (0.44) | 4.78 (1.94) | 4.80 (1.81) |
| Chair | 3.41 (1.51) | 3.17 (1.02) | 5.49 (1.26) | 5.04 (1.60) |
| Chicken | 1.36 (0.63) | 1.22 (0.55) | 3.89 (1.47) | 5.46 (1.65) |
| Cicada | 3.75 (1.55) | 3.34 (1.13) | 5.52 (1.53) | 5.08 (1.71) |
| Clearing throat | 1.27 (0.56) | 1.32 (0.53) | 5.59 (1.19) | 5.99 (1.71) |
| Clock | 2.69 (1.49) | 2.52 (1.19) | 5.45 (1.23) | 5.61 (1.95) |
| Coffee machine | 3.36 (1.34) | 3.11 (1.07) | 5.52 (1.33) | 5.10 (1.74) |
| Coin | 1.99 (1.00) | 1.93 (0.85) | 4.57 (1.20) | 5.48 (1.77) |
| Computer | 3.32 (1.33) | 3.02 (0.93) | 5.46 (1.33) | 5.20 (1.62) |
| Cow | 1.54 (0.88) | 1.46 (0.76) | 3.85 (1.41) | 5.71 (1.51) |
| Cricket | 1.62 (0.70) | 1.54 (0.68) | 4.08 (1.56) | 6.46 (1.74) |
| Crow | 1.87 (0.88) | 1.74 (0.77) | 5.43 (1.55) | 5.28 (1.59) |
| Cutlery | 2.89 (1.36) | 2.70 (1.09) | 5.18 (1.05) | 5.44 (1.61) |
| Dog barking | 1.30 (0.64) | 1.45 (0.75) | 4.66 (1.78) | 4.41 (1.77) |
| Donkey | 2.60 (1.44) | 2.16 (1.16) | 4.68 (1.50) | 5.50 (1.53) |
| Door | 2.48 (1.42) | 2.32 (1.19) | 5.32 (1.13) | 5.51 (1.55) |
| Doorknob | 2.85 (1.27) | 2.72 (1.01) | 5.03 (1.06) | 5.82 (1.65) |
| Drill | 2.35 (1.10) | 2.28 (0.89) | 5.56 (1.42) | 4.74 (1.71) |
| Drum | 1.54 (0.69) | 1.41 (0.61) | 3.03 (1.24) | 4.61 (1.93) |
| Duck | 2.17 (1.01) | 2.07 (0.84) | 4.47 (1.40) | 5.65 (1.62) |
| Elephant | 1.87 (0.96) | 1.69 (0.92) | 4.26 (1.56) | 4.51 (1.82) |
| Fire | 2.71 (1.23) | 2.51 (1.10) | 5.50 (1.77) | 4.78 (1.88) |
| Fire alarm | 1.29 (0.58) | 1.15 (0.38) | 6.61 (1.30) | 3.21 (1.56) |
| Fire truck | 2.15 (1.26) | 2.00 (1.02) | 6.54 (1.25) | 3.57 (1.44) |
| Flute | 2.02 (1.04) | 1.68 (0.68) | 3.19 (1.30) | 6.02 (1.85) |
| Fly | 1.87 (0.96) | 1.83 (0.85) | 6.37 (1.56) | 4.46 (1.56) |
| Footsteps | 2.24 (1.07) | 2.20 (0.97) | 4.68 (0.95) | 5.34 (1.46) |
| Frog | 1.41 (0.60) | 1.32 (0.53) | 4.09 (1.58) | 5.89 (1.78) |
| Gargle | 1.93 (0.98) | 1.77 (0.85) | 4.88 (1.22) | 6.13 (1.53) |
| Goat | 2.04 (1.13) | 1.73 (0.84) | 4.07 (1.54) | 5.53 (1.68) |
| Gong | 1.91 (1.00) | 1.56 (0.83) | 3.94 (1.39) | 4.38 (1.78) |
| Goose | 2.48 (1.11) | 2.25 (0.90) | 4.59 (1.24) | 5.24 (1.72) |
| Grasshopper | 2.68 (1.24) | 2.47 (0.96) | 4.74 (1.41) | 6.06 (1.58) |
| Guitar | 1.49 (0.71) | 1.42 (0.60) | 2.83 (1.36) | 4.75 (2.03) |
| Helicopter | 1.71 (0.72) | 1.47 (0.63) | 4.51 (1.39) | 4.23 (1.75) |
| Horse | 1.27 (0.53) | 1.08 (0.30) | 3.88 (1.58) | 4.24 (1.74) |
| Keys | 2.89 (1.29) | 2.81 (1.07) | 5.07 (1.05) | 5.54 (1.52) |
| Knife | 3.09 (1.26) | 3.04 (1.00) | 5.02 (1.14) | 5.41 (1.56) |
| Kookaburra | 1.93 (1.19) | 1.80 (0.88) | 4.04 (1.69) | 4.89 (1.68) |
| Laugh | 1.15 (0.36) | 1.29 (0.58) | 2.32 (1.11) | 4.54 (2.06) |
| Lighter | 3.35 (1.41) | 3.00 (1.08) | 5.54 (1.29) | 5.69 (1.55) |
| Lion | 2.42 (1.23) | 2.11 (0.96) | 5.31 (1.50) | 4.08 (1.61) |
| Machine gun | 2.14 (1.06) | 1.76 (0.80) | 6.95 (1.61) | 3.22 (1.78) |
| Maracas | 2.72 (1.09) | 2.41 (0.85) | 4.02 (1.29) | 5.67 (1.74) |
| Match | 2.64 (1.32) | 2.54 (1.11) | 5.10 (1.24) | 5.34 (1.61) |
| Microwave | 2.75 (1.36) | 2.69 (1.05) | 4.84 (1.20) | 5.92 (1.62) |
| Monkey | 1.74 (1.04) | 1.43 (0.68) | 3.66 (1.60) | 4.91 (1.66) |
| Mosquito | 2.34 (1.31) | 2.31 (1.13) | 6.80 (1.45) | 4.25 (1.85) |
| Mouse | 2.36 (1.12) | 2.16 (0.89) | 4.53 (1.81) | 5.60 (1.64) |
| Music box | 1.85 (0.78) | 1.76 (0.67) | 3.10 (1.21) | 6.60 (1.84) |
| Noisy miner | 2.94 (1.18) | 2.50 (0.92) | 4.85 (1.57) | 4.73 (1.52) |
| Ocean | 2.46 (1.12) | 2.41 (1.01) | 3.95 (1.62) | 5.59 (1.96) |
| Owl | 2.02 (1.07) | 1.77 (0.96) | 4.00 (1.41) | 5.89 (1.87) |
| Paper | 2.61 (1.14) | 2.63 (0.99) | 4.79 (1.17) | 5.96 (1.75) |
| Parrot | 2.02 (0.95) | 1.98 (0.90) | 4.88 (1.54) | 5.01 (1.74) |
| Pen | 2.89 (1.41) | 2.81 (1.10) | 5.06 (1.14) | 5.61 (1.65) |
| Phone | 1.19 (0.50) | 1.17 (0.46) | 4.14 (1.52) | 4.89 (1.79) |
| Piano | 1.25 (0.51) | 1.26 (0.49) | 2.80 (1.37) | 5.41 (2.08) |
| Pig | 2.82 (1.41) | 2.66 (1.30) | 4.74 (1.34) | 5.69 (1.64) |
| Pigeon | 2.10 (0.94) | 1.94 (0.89) | 4.53 (1.46) | 6.06 (1.72) |
| Pinball machine | 3.28 (1.40) | 3.03 (1.13) | 4.76 (1.42) | 5.08 (1.67) |
| Plane | 2.07 (1.01) | 2.13 (0.98) | 4.59 (1.59) | 4.28 (1.82) |
| Plates | 3.78 (1.56) | 3.23 (1.14) | 5.07 (1.10) | 5.59 (1.40) |
| Printer | 3.07 (1.31) | 2.90 (1.03) | 5.06 (1.13) | 5.64 (1.58) |
| Radio | 2.28 (1.27) | 2.24 (1.07) | 4.72 (1.13) | 5.50 (1.64) |
| Rain | 1.54 (0.70) | 1.69 (0.81) | 3.41 (1.58) | 6.22 (2.01) |
| River | 1.76 (0.79) | 1.68 (0.81) | 3.18 (1.40) | 6.42 (1.96) |
| Rock fall | 3.44 (1.40) | 3.23 (1.07) | 5.11 (1.12) | 5.27 (1.55) |
| Rooster | 1.41 (0.75) | 1.34 (0.69) | 4.39 (1.64) | 4.62 (1.79) |
| Saxophone | 1.54 (0.82) | 1.36 (0.59) | 3.14 (1.43) | 5.15 (2.00) |
| Scissors | 3.41 (1.34) | 3.22 (1.07) | 5.36 (1.27) | 5.82 (1.64) |
| Seal | 3.41 (1.42) | 2.72 (1.18) | 4.67 (1.55) | 5.28 (1.53) |
| Sheep | 1.55 (0.78) | 1.46 (0.75) | 3.84 (1.31) | 5.56 (1.66) |
| Shower | 3.32 (1.52) | 2.94 (1.15) | 5.16 (1.43) | 5.58 (1.59) |
| Skiing | 3.86 (1.36) | 3.45 (0.99) | 5.43 (1.24) | 5.39 (1.64) |
| Sneeze | 1.09 (0.31) | 1.15 (0.49) | 4.83 (1.49) | 4.96 (1.72) |
| Snore | 1.87 (1.09) | 1.90 (1.04) | 5.50 (1.72) | 5.37 (1.73) |
| Tambourine | 2.12 (1.02) | 1.95 (0.95) | 3.87 (1.40) | 5.33 (1.70) |
| Tennis | 3.80 (1.56) | 3.39 (1.08) | 5.13 (1.37) | 5.30 (1.82) |
| Toaster | 3.69 (1.45) | 3.40 (1.01) | 5.24 (1.29) | 5.71 (1.69) |
| Toilet | 1.94 (1.29) | 2.06 (1.13) | 5.11 (1.02) | 5.98 (1.48) |
| Train | 2.72 (1.36) | 2.61 (1.08) | 4.83 (1.51) | 5.32 (1.71) |
| Triangle | 1.98 (0.90) | 1.86 (0.82) | 4.21 (1.34) | 5.20 (1.63) |
| Trumpet | 1.68 (0.82) | 1.49 (0.69) | 3.37 (1.29) | 4.90 (2.08) |
| Turkey | 2.45 (1.31) | 2.07 (1.11) | 4.47 (1.45) | 5.30 (1.66) |
| Vacuum cleaner | 2.35 (1.12) | 2.23 (0.99) | 5.34 (1.25) | 4.89 (1.68) |
| Washing machine | 3.33 (1.42) | 3.13 (1.07) | 5.59 (1.23) | 5.81 (1.75) |
| Water bubbling | 2.15 (1.04) | 2.10 (0.94) | 4.02 (1.38) | 5.84 (1.52) |
| Whale | 3.61 (1.47) | 3.11 (1.09) | 5.43 (1.61) | 4.72 (2.03) |
| Whip | 2.72 (1.33) | 2.52 (1.09) | 5.19 (1.48) | 4.72 (1.66) |
| Whistle | 1.59 (0.64) | 1.55 (0.69) | 4.64 (1.44) | 4.18 (1.79) |
| Whistling | 1.38 (0.62) | 1.42 (0.63) | 3.71 (1.70) | 5.50 (1.87) |
| Wind | 2.76 (1.39) | 2.56 (1.17) | 5.37 (1.52) | 4.80 (1.78) |
| Wind chime | 2.05 (0.93) | 1.91 (0.81) | 3.93 (1.59) | 6.03 (1.81) |
| Wolf | 2.11 (1.06) | 1.98 (0.91) | 5.29 (1.54) | 4.53 (1.85) |
| Yawn | 1.83 (1.01) | 2.07 (1.04) | 4.85 (1.35) | 6.76 (1.58) |
