# Supplementary material for: NESSTI: Norms for Environmental Sound Stimuli
Source: PLoS One. 2013 Sep 4;8(9):e73382. doi: 10.1371/journal.pone.0073382 (PMC3762767; doi:10.1371/journal.pone.0073382)
Supplement: Table S4 — Classification and variable ratings by category for sounds in Study 1. (DOCX) [file pone.0073382.s004.docx]

**Supporting Information**

**Table S4. Correct classification by category and variable ratings by category**

| **Category** | **N** | **Classification**  **Mean (SD)** | **Familiarity**  **Mean (SD)** | **Representativeness Mean (SD)** | **Pleasantness**  **Mean (SD)** | **Arousal**  **Mean (SD)** |
| --- | --- | --- | --- | --- | --- | --- |
| **Alarm** | 2 | 57.72 (36.79) | 1.44 (0.21) | 1.35 (0.29) | 5.63 (1.39) | 3.70 (0.68) |
| **Animal** | 38 | 87.16 (16.73) | 2.18 (0.68) | 1.98 (0.58) | 4.68 (0.77) | 5.18 (0.63) |
| **Household** | 27 | 76.26 (13.90) | 2.84 (0.59) | 2.67 (0.49) | 5.15 (0.34) | 5.49 (0.33) |
| **Human** | 10 | 88.46 (16.22) | 1.72 (0.50) | 1.72 (0.43) | 4.77 (1.06) | 5.45 (0.74) |
| **Musical** | 12 | 85.09 (19.88) | 1.85 (0.39) | 1.67 (0.33) | 3.45 (0.51) | 5.34 (0.65) |
| **Nature** | 7 | 64.81 (23.29) | 2.40 (0.65) | 2.31 (0.54) | 4.36 (0.95) | 5.56 (0.65) |
| **Recreational** | 5 | 25.37 (15.90) | 3.34 (0.72) | 3.04 (0.60) | 4.93 (0.55) | 5.30 (0.21) |
| **Transport** | 6 | 69.32 (35.08) | 2.14 (0.44) | 2.04 (0.46) | 5.15 (0.95) | 4.38 (0.78) |
| **Weapon** | 3 | 63.12 (27.67) | 2.59 (0.40) | 2.23 (0.41) | 6.14 (0.89) | 3.92 (0.76) |
| **Total** | 110 | 77.98 (22.94) | 2.33 (0.73) | 2.16 (0.64) | 4.74 (0.89) | 5.22 (0.69) |
