# Supplementary material for: NESSTI: Norms for Environmental Sound Stimuli
Source: PLoS One. 2013 Sep 4;8(9):e73382. doi: 10.1371/journal.pone.0073382 (PMC3762767; doi:10.1371/journal.pone.0073382)
Supplement: Table S5 — Sound identification data and related response measures for each sound in Study 2. (DOCX) [file pone.0073382.s005.docx]

**Supporting Information**

**Table S5. Study 2 sounds listed in order of identification accuracy with proportion correct, modal response, H-value, mean reaction time in milliseconds, confidence and imageability ratings.**

| **Sound** | **Number of Correct Responses** | **ID % Correct** | **% Modal Response** | **H-Value** | **Reaction Time Mean (SD)** | **Confidence Mean (SD)** | **Imageability**  **Mean (SD)** |
| --- | --- | --- | --- | --- | --- | --- | --- |
| Horse | 53 | 100.00 | 98.11 | 0.14 | 1633 (325) | 6.72 (0.97) | 8.34 (1.51) |
| Dog barking (MR: Dog) | 53 | 100.00 | 71.70 | 1.22 | 1715 (359) | 6.75 (1.00) | 8.34 (1.48) |
| Piano | 52 | 98.11 | 94.23 | 0.37 | 1800 (531) | 6.53 (0.91) | 8.19 (1.48) |
| Baby crying (MR: Baby) | 52 | 98.11 | 71.15 | 0.97 | 1902 (400) | 6.42 (1.06) | 7.92 (1.84) |
| Helicopter | 50 | 98.04 | 98.00 | 0.14 | 1667 (557) | 6.11 (1.72) | 7.77 (2.14) |
| Laugh | 51 | 96.23 | 100.00 | 0.00 | 1564 (381) | 6.70 (1.05) | 8.13 (1.57) |
| Cow | 50 | 96.15 | 98.00 | 0.14 | 1993 (631) | 6.36 (1.35) | 8.00 (1.48) |
| Guitar | 50 | 96.15 | 96.00 | 0.24 | 1823 (431) | 6.68 (0.70) | 8.30 (1.19) |
| Phone | 50 | 96.15 | 88.00 | 0.68 | 1777 (460) | 6.75 (0.71) | 8.38 (1.00) |
| River | 50 | 96.15 | 76.00 | 1.42 | 1825 (485) | 6.55 (0.82) | 8.21 (1.08) |
| Coin | 50 | 96.15 | 74.00 | 1.38 | 1969 (475) | 5.77 (1.69) | 7.49 (1.91) |
| Toilet | 48 | 96.00 | 56.25 | 1.30 | 1914 (567) | 5.94 (1.56) | 7.49 (2.03) |
| Door | 47 | 95.92 | 68.09 | 1.63 | 2398 (527) | 5.47 (1.67) | 6.89 (2.15) |
| Frog | 49 | 94.23 | 95.92 | 0.29 | 1703 (481) | 6.49 (1.25) | 8.23 (1.30) |
| Cat | 49 | 92.45 | 95.92 | 0.25 | 1593 (566) | 6.83 (0.58) | 8.51 (1.33) |
| Sneeze | 47 | 92.16 | 100.00 | 0.00 | 1264 (357) | 6.49 (1.45) | 8.08 (1.91) |
| Whistling | 47 | 92.16 | 100.00 | 0.00 | 1712 (343) | 6.49 (1.33) | 7.57 (2.20) |
| Drum | 47 | 88.68 | 85.11 | 0.88 | 2019 (590) | 6.28 (1.29) | 7.51 (2.14) |
| Snore (MR: Snoring) | 45 | 88.24 | 88.89 | 0.58 | 2146 (562) | 5.87 (1.80) | 6.83 (2.35) |
| Bicycle (MR: Bell) | 43 | 87.76 | 46.51 | 1.57 | 2619 (615) | 5.06 (1.77) | 5.98 (2.63) |
| Owl | 46 | 86.79 | 97.83 | 0.15 | 1643 (414) | 6.43 (1.05) | 7.89 (1.70) |
| Canary (MR: Bird) | 46 | 86.79 | 84.78 | 0.94 | 1889 (527) | 6.66 (0.55) | 7.96 (1.26) |
| Paper | 43 | 86.00 | 60.47 | 2.03 | 2257 (686) | 5.49 (1.54) | 6.68 (2.29) |
| Budgie (MR: Birds) | 43 | 84.31 | 83.72 | 0.88 | 2161 (605) | 6.00 (1.52) | 6.89 (2.35) |
| Crow | 44 | 83.02 | 50.00 | 1.40 | 2314 (659) | 6.25 (1.16) | 7.43 (1.81) |
| Gargle (MR: Gargling) | 43 | 82.69 | 69.77 | 1.33 | 1834 (546) | 6.11 (1.60) | 7.57 (1.85) |
| Clearing Throat (MR: Cough) | 42 | 82.35 | 45.24 | 1.71 | 2504 (729) | 6.13 (1.23) | 7.30 (2.00) |
| Rooster | 40 | 81.63 | 80.00 | 0.98 | 2322 (800) | 5.91 (1.66) | 7.53 (2.05) |
| Yawn | 43 | 81.13 | 100.00 | 0.00 | 1766 (405) | 6.21 (1.55) | 7.92 (1.65) |
| Duck | 43 | 81.13 | 53.49 | 1.26 | 2174 (655) | 6.09 (1.36) | 7.40 (1.92) |
| Chicken | 42 | 80.77 | 83.33 | 0.79 | 2205 (738) | 6.30 (1.46) | 8.06 (1.75) |
| Fire | 41 | 78.85 | 85.37 | 0.86 | 1915 (648) | 5.70 (1.54) | 7.34 (2.10) |
| Wolf | 40 | 78.43 | 85.00 | 0.7476 | 2101 (604) | 6.09 (1.26) | 7.75 (1.91) |
| Plane | 38 | 77.55 | 89.47 | 0.64 | 1993 (440) | 5.13 (1.90) | 6.40 (2.47) |
| Water Bubbling (MR: Bubbles) | 38 | 76.00 | 42.11 | 2.20 | 2231 (555) | 5.09 (1.68) | 6.47 (2.30) |
| Trumpet | 37 | 75.51 | 100.00 | 0.00 | 1966 (520) | 5.89 (1.52) | 6.94 (2.37) |
| Footsteps (MR: Walking) | 37 | 75.51 | 45.95 | 1.98 | 2200 (468) | 5.30 (1.69) | 6.08 (2.39) |
| Car | 40 | 75.47 | 62.50 | 2.00 | 2011 (478) | 6.45 (1.12) | 7.98 (1.55) |
| Whip | 30 | 75.00 | 90.00 | 0.63 | 1910 (474) | 4.81 (2.18) | 5.58 (2.91) |
| Wind | 36 | 73.47 | 83.33 | 0.9697 | 2191 (584) | 4.98 (1.86) | 5.42 (2.83) |
| Broom (MR: Sweeping) | 33 | 71.74 | 57.58 | 1.59 | 2297 (758) | 4.64 (1.93) | 5.75 (2.79) |
| Whistle | 37 | 71.15 | 91.89 | 0.48 | 2015 (534) | 5.77 (1.54) | 6.72 (1.95) |
| Parrot (MR: Bird) | 34 | 70.83 | 85.29 | 0.94 | 2466 (586) | 5.25 (1.73) | 5.49 (2.52) |
| Noisy miner (MR: Bird) | 34 | 69.39 | 100.00 | 0.00 | 2622 (710) | 4.79 (1.93) | 5.70 (2.72) |
| Donkey | 30 | 68.18 | 100.00 | 0.00 | 2337 (599) | 5.49 (2.02) | 6.47 (2.67) |
| Tambourine | 32 | 68.09 | 93.75 | 0.34 | 2211 (700) | 5.96 (1.37) | 7.32 (2.01) |
| Fire Alarm (MR: Siren) | 36 | 67.92 | 63.89 | 1.60 | 1709 (562) | 6.55 (1.14) | 7.49 (2.14) |
| Radio | 35 | 67.31 | 91.43 | 0.56 | 2186 (586) | 5.94 (1.22) | 7.06 (1.93) |
| Machine Gun (MR: Gun) | 35 | 67.31 | 57.14 | 2.08 | 2373 (690) | 5.49 (1.61) | 6.38 (2.40) |
| Cricket | 34 | 66.67 | 97.06 | 0.19 | 2008 (635) | 6.09 (1.17) | 7.26 (1.89) |
| Sheep | 33 | 64.71 | 100.00 | 0.00 | 2260 (771) | 5.87 (1.69) | 7.17 (2.10) |
| Saxophone | 33 | 63.46 | 84.85 | 0.72 | 2140 (584) | 6.30 (0.97) | 7.98 (1.25) |
| Kookaburra | 32 | 62.75 | 71.88 | 0.86 | 2029 (515) | 5.75 (1.90) | 6.96 (2.39) |
| Elephant | 30 | 62.50 | 100.00 | 0.00 | 2136 (849) | 5.00 (2.10) | 6.28 (2.76) |
| Doorknob (MR: Door) | 31 | 62.00 | 64.52 | 1.54 | 2443 (756) | 4.91 (1.70) | 5.66 (2.27) |
| Lighter | 29 | 61.70 | 65.52 | 1.66 | 2265 (790) | 5.02 (2.14) | 6.55 (2.77) |
| Wind Chimes | 30 | 61.22 | 53.33 | 0.9968 | 2055 (496) | 5.60 (1.80) | 6.87 (2.63) |
| Turkey | 30 | 60.00 | 60.00 | 1.14 | 2084 (644) | 5.55 (1.78) | 6.45 (2.29) |
| Bee | 29 | 59.18 | 93.10 | 0.43 | 2095 (665) | 5.87 (1.58) | 7.19 (2.47) |
| Gong | 27 | 58.70 | 100.00 | 0.00 | 1889 (594) | 5.81 (1.77) | 7.57 (2.00) |
| Train | 27 | 58.70 | 96.30 | 0.23 | 2213 (700) | 4.58 (1.90) | 5.45 (2.81) |
| Pigeon (MR: Bird) | 30 | 56.60 | 56.67 | 1.55 | 2733 (787) | 5.57 (1.60) | 6.32 (2.52) |
| Flute | 26 | 56.52 | 88.46 | 0.52 | 2129 (603) | 5.58 (1.68) | 6.17 (2.40) |
| Lion | 27 | 56.25 | 88.89 | 0.50 | 2223 (690) | 5.36 (1.65) | 6.77 (2.11) |
| Fire Truck (MR: Siren) | 27 | 56.25 | 55.56 | 2.06 | 1318 (817) | 4.74 (2.07) | 5.77 (2.92) |
| Mosquitos | 28 | 54.90 | 85.71 | 0.71 | 2277 (475) | 5.06 (2.00) | 6.47 (2.56) |
| Match | 25 | 53.19 | 52.00 | 1.63 | 2434 (687) | 4.72 (1.73) | 5.51 (2.64) |
| Monkey | 26 | 50.98 | 84.62 | 0.74 | 2172 (660) | 6.19 (1.14) | 7.26 (2.25) |
| Maracas (MR: Maracas/Shaker) | 22 | 50.00 | 40.91 | 1.77 | 2433 (688) | 4.43 (2.10) | 5.51 (2.95) |
| Burp | 21 | 48.84 | 90.48 | 0.55 | 2298 (550) | 4.98 (1.97) | 5.68 (2.83) |
| Clock | 22 | 47.83 | 59.09 | 1.75 | 2158 (663) | 4.32 (2.37) | 5.30 (3.07) |
| Cutlery | 20 | 45.45 | 70.00 | 1.56 | 2595 (736) | 4.15 (1.94) | 5.17 (2.73) |
| Vacuum cleaner | 24 | 45.28 | 54.17 | 1.00 | 2068 (622) | 4.85 (1.82) | 5.70 (2.46) |
| Shower (MR: Water) | 19 | 45.24 | 63.16 | 1.98 | 2707 (710) | 3.64 (1.99) | 4.64 (2.91) |
| Pen | 19 | 45.24 | 47.37 | 2.22 | 2573 (516) | 3.74 (2.09) | 4.68 (3.05) |
| Rain | 23 | 45.10 | 86.96 | 0.77 | 1810 (395) | 5.49 (1.90) | 6.85 (2.48) |
| Microwave | 21 | 44.68 | 100.00 | 0.00 | 2142 (472) | 5.23 (1.96) | 5.91 (2.78) |
| Computer | 21 | 43.75 | 80.95 | 0.86 | 2751 (649) | 4.06 (1.74) | 4.72 (2.39) |
| Keys | 20 | 42.55 | 70.00 | 0.88 | 2235 (480) | 4.28 (2.04) | 5.49 (2.76) |
| Chair | 17 | 42.50 | 52.94 | 2.29 | 2278 (454) | 3.81 (1.90) | 4.47 (2.85) |
| Goose (MR: Bird) | 22 | 42.31 | 63.64 | 1.33 | 2587 (641) | 5.4 (1.52) | 6.58 (2.25) |
| Music Box | 18 | 39.13 | 72.22 | 1.23 | 2530 (773) | 5.28 (1.93) | 7.13 (2.42) |
| Fly | 20 | 38.46 | 90.00 | 0.57 | 2279 (586) | 6.09 (1.46) | 7.51 (1.93) |
| Pig | 15 | 34.09 | 86.67 | 0.57 | 2171 (467) | 4.28 (2.32) | 5.06 (3.13) |
| Cannon (MR: Bomb) | 14 | 30.43 | 64.29 | 1.20 | 2222 (671) | 4.77 (2.04) | 6.04 (2.79) |
| Cicada (MR: Insects) | 11 | 29.73 | 63.64 | 1.24 | 3146 (649) | 3.08 (1.98) | 3.45 (2.63) |
| Goat | 14 | 28.57 | 100.00 | 0.00 | 2338 (644) | 5.55 (1.85) | 6.42 (2.44) |
| Bat (MR: Flying) | 9 | 23.08 | 55.56 | 1.44 | 2625 (562) | 3.77 (2.17) | 4.26 (3.05) |
| Drill | 9 | 22.50 | 100.00 | 0.00 | 2103 (570) | 4.25 (2.26) | 4.79 (3.00) |
| Tennis | 8 | 20.00 | 62.50 | 1.55 | 2444 (974) | 3.23 (1.84) | 4.02 (2.76) |
| Triangle | 10 | 19.23 | 90.00 | 0.47 | 2220 (497) | 5.94 (1.54) | 7.13 (2.26) |
| Ocean (MR: Wave) | 8 | 17.39 | 50.00 | 1.50 | 2241 (815) | 4.17 (1.94) | 5.23 (2.58) |
| Seal | 6 | 15.38 | 100.00 | 0.00 | 2581 (380) | 3.21 (2.01) | 3.72 (2.71) |
| Plates (MR: Dishes) | 6 | 13.95 | 50.00 | 1.79 | 3236 (895) | 3.79 (1.96) | 4.26 (2.69) |
| Pinball (MR: Pinball/Pinball machine) | 6 | 13.64 | 50.00 | 1.00 | 2648 (539) | 3.75 (2.17) | 4.60 (2.96) |
| Grasshopper (MR: Insect) | 6 | 11.54 | 100.00 | 0.00 | 2293 (802) | 5.30 (1.72) | 6.21 (2.54) |
| Printer (Photocopier) | 4 | 10.53 | 75.00 | 0.81 | 2591 (455) | 3.81 (2.17) | 4.00 (2.90) |
| Rock Fall (MR: Rocks/Rockslide/Rubble/ Stones dropping) | 4 | 9.52 | 25.00 | 2.00 | 3476 (247) | 3.34 (1.88) | 4.13 (2.73) |
| Knife (MR: Chopping/Chopping wood/ Cutting/Cutting board) | 4 | 9.09 | 25.00 | 2.00 | 3186 (1004) | 3.68 (1.84) | 4.51 (2.67) |
| Basketball (MR: Ball/Basketball/Bouncing ball/Dribbling basketball) | 4 | 7.69 | 25.00 | 2.00 | 2327 (219) | 5.79 (1.39) | 6.64 (2.08) |
| Book ( MR: Book/Moving paper) | 2 | 6.06 | 50.00 | 1.00 | 2562 (149) | 2.87 (2.22) | 3.06 (2.58) |
| Bear | 2 | 5.13 | 100.00 | 0.00 | 2544 (453) | 4.32 (2.11) | 4.77 (3.02) |
| Scissors ( MR: Scissors/Clip) | 2 | 5.13 | 50.00 | 1.00 | 3272 (1597) | 3.09 (1.76) | 3.40 (2.40) |
| Skiing (MR: Ice/Sliding) | 2 | 5.00 | 50.00 | 1.00 | 3265 (111) | 3.57 (2.21) | 4.23 (2.95) |
| Mouse | 2 | 3.85 | 100.00 | 0.00 | 2101 (464) | 5.79 (1.35) | 7.30 (1.83) |
| Whale ( MR: Marine life) | 1 | 2.78 | 100.00 | 0.00 | 2727 | 3.40 (2.20) | 3.83 (2.95) |
| Bull | 0 | 0.00 | - | - | - | 6.11 (1.60) | 7.66 (1.95) |
| Coffee Machine | 0 | 0.00 | - | - | - | 4.60 (1.92) | 5.06 (2.65) |
| Toaster | 0 | 0.00 | - | - | - | 2.98 (1.94) | 3.72 (2.87) |
| Washing Machine | 0 | 0.00 | - | - | - | 4.08 (1.95) | 4.49 (2.62) |

Key: MR = Modal Response. NB H-Value and reaction times based on correct responses only.
