# Supplementary figures and images for: NESSTI: Norms for Environmental Sound Stimuli
Source: PLoS One. 2013 Sep 4;8(9):e73382. doi: 10.1371/journal.pone.0073382 (PMC3762767; doi:10.1371/journal.pone.0073382)

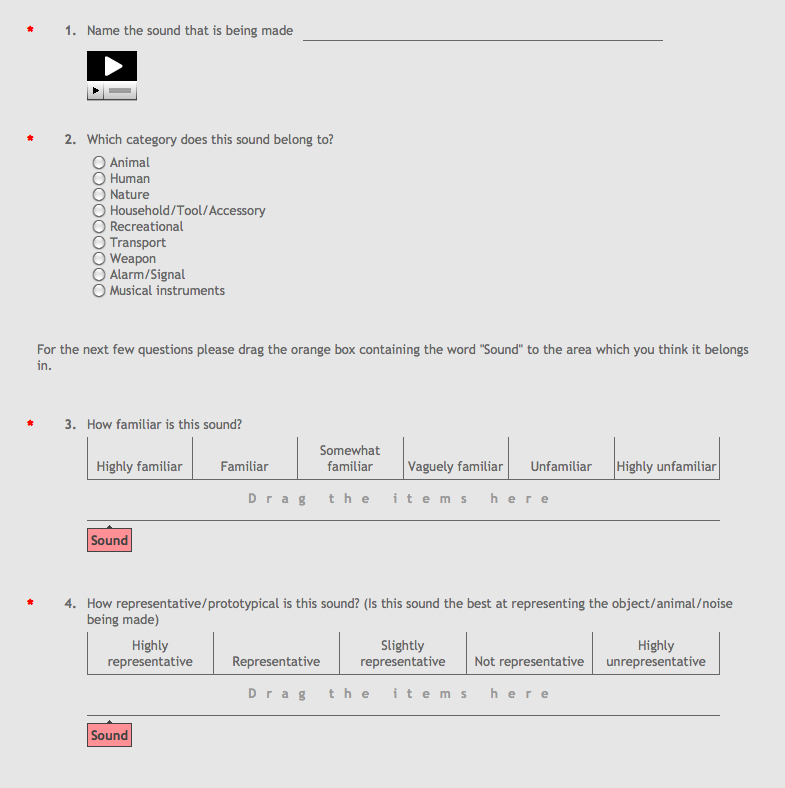


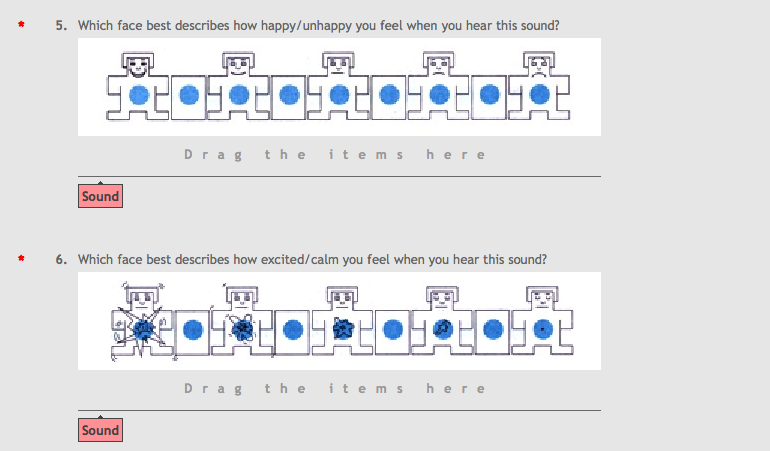

Supplement: Questionnaire S1 — A screenshot of the online questionnaire used to probe the participants’ knowledge for each environmental sound. (DOCX) [file pone.0073382.s006.docx]
